# Learning-induced mRNA alterations in olfactory bulb mitral cells in neonatal rats

**DOI:** 10.1101/lm.051177.119

**Published:** 2020-05

**Authors:** Michaelina N. Nartey, Lourdes Peña-Castillo, Megan LeGrow, Jules Doré, Sriya Bhattacharya, Andrea Darby-King, Samantha J. Carew, Qi Yuan, Carolyn W. Harley, John H. McLean

**Affiliations:** 1 Divison of Biomedical Sciences, Memorial University of Newfoundland, St. John's, Newfoundland A1B3V6, Canada; 2 Department of Computer Science, Memorial University of Newfoundland, St. John's, Newfoundland A1B3X5, Canada; 3 Department of Psychology, Memorial University of Newfoundland, St. John's, Newfoundland A1B3X9, Canada

## Abstract

In the olfactory bulb, a cAMP/PKA/CREB-dependent form of learning occurs in the first week of life that provides a unique mammalian model for defining the epigenetic role of this evolutionarily ancient plasticity cascade. Odor preference learning in the week-old rat pup is rapidly induced by a 10-min pairing of odor and stroking. Memory is demonstrable at 24 h, but not 48 h, posttraining. Using this paradigm, pups that showed peppermint preference 30 min posttraining were sacrificed 20 min later for laser microdissection of odor-encoding mitral cells. Controls were given odor only. Microarray analysis revealed that 13 nonprotein-coding mRNAs linked to mRNA translation and splicing and 11 protein-coding mRNAs linked to transcription differed with odor preference training. MicroRNA23b, a translation inhibitor of multiple plasticity-related mRNAs, was down-regulated. Protein-coding transcription was up-regulated for Sec23b, Clic2, Rpp14, Dcbld1, Magee2, Mstn, Fam229b, RGD1566265, and Mgst2. Gng12 and Srcg1 mRNAs were down-regulated. Increases in Sec23b, Clic2, and Dcbld1 proteins were confirmed in mitral cells in situ at the same time point following training. The protein-coding changes are consistent with extracellular matrix remodeling and ryanodine receptor involvement in odor preference learning. A role for CREB and AP1 as triggers of memory-related mRNA regulation is supported. The small number of gene changes identified in the mitral cell input/output link for 24 h memory will facilitate investigation of the nature, and reversibility, of changes supporting temporally restricted long-term memory.

Early odor preference learning in the rat pup is an exceptional model for illuminating the epigenetic changes that support learning and memory. Early odor preference learning reflects the operation of an evolutionarily ancient associative plasticity cascade expressed in mammals as well as invertebrates. That cascade, cAMP/PKA/CREB, was first illuminated in *Aplysia* (Kandel 2012), and is triggered in the mollusk by serotonin. Kandel proposed that in mammals (Brunelli et al. 1976), the same plasticity mechanisms would be engaged by norepinephrine (NE). Forebrain NE release by locus coeruleus neurons occurs with tactile activation in rat pups (Kimura and Nakamura 1985; Nakamura et al. 1987). NE then acts through β-adrenergic receptors in the olfactory bulb (OB) to generate a pulsatile cAMP wave (Cui et al. 2007) that provides the unconditioned stimulus (US) for odor preference learning. In this model, β-adrenergic activation is both necessary and sufficient for odor preference memory (Sullivan et al. 1989). Glutamatergic olfactory nerve input to OB mitral cells carries the conditioned stimulus (CS) (Cui et al. 2011). Pairing CS and US in the first week of life generates odor preferences that allow pups to maintain proximity to their dams. A single 10 min odor paired with stroking or optimal β-adrenergic activation activates CREB (McLean et al. 1999), but produces only 24 h memory (McLean et al. 2005), while repeated pairings of odor and maternal care can generate life-long memories (Fillion and Blass 1986; Shah et al. 2002). Thus, the model can also provide a tool for exploring the epigenetic underpinning of memory duration.

Importantly, the changes critical for expression of odor preference memory occur in topographically organized odor-responsive mitral cells, which both receive odor nerve input to the OB and then transmit the primary OB output to cortical structures. Since mitral cells have topographical organization, unlike representations in more complex structures such as hippocampus, these cellular transducers of odor memories can be isolated using laser microdissection (LMD).

At later developmental stages, the US is no longer a simple function of OB β-adrenergic activation and OB mitral cell changes. Thus, the first week of life provides a singular opportunity to probe evolutionarily ancient biological memory mechanisms underlying a well-characterized associative memory in the mammalian nervous system.

The early odor preference learning and memory model reflects four classes of changes linked to memory expression as reviewed in detail below: (1) increased AMPA receptor (AMPAR) density and current for the trained odor input permitting robust activation by that odor; (2) decreased NMDA receptor (NMDAR) 2b subunits suggesting reduced plasticity and increased stability; (3) increased metabolic activity in the encoding region, permitting more active circuits; and consistent with the foregoing, (4) increased representational stability as indexed by *arc* catFish, of both excitatory and inhibitory peppermint cell activation in the mitral and granule cells, respectively. This odor preference learning model shares conserved features of learning and memory previously identified in species from fruit flies to humans (Kandel 2012).

Novel glutamatergic odor input was provided from peppermint-scented bedding. This CS activates mitral cell AMPA and NMDA receptors. A single 10 min training trial that pairs a novel odor with stroking leads to a learned odor preference readily demonstrated at 24 h, but not at 48 h (for a recent review of the model see Yuan et al. 2014).

This 24 h odor preference long-term memory (LTM) requires protein synthesis. Blocking mRNA transcription in the first hour posttraining, but not after, prevents 24 h LTM, while blocking mRNA translation in the first hour, but not after, prevents both intermediate-term memory (∼5 h) and 24 h LTM. Short-term memory for odor preference (<3 h) does not require protein synthesis (Grimes et al. 2011).

Multiple intracellular signaling changes associated with memory initiation have been characterized. Ten minutes following training that produces a 24 h peppermint odor preference, cAMP (Cui et al. 2007), PKA activity (Grimes et al. 2012), AMPAR GluA1 subunit phosphorylation (Cui et al. 2011), and CREB phosphorylation (McLean et al. 1999) are at their highest levels, subsequently declining back to baseline. Blocking bulbar pCREB production prevents early odor preference learning (Yuan et al. 2003). Glomerular NMDAR phosphorylation on the PKA-sensitive GluN1 phosphorylation site is highest at the first time point examined, 5 min posttraining (Lethbridge et al. 2012). Peppermint odor is represented in the dorsolateral and dorsomedial quadrants of the OB, as previously indexed by metabolic markers (e.g, Sullivan and Leon 1986) and pCREB increases (McLean et al. 1999).

Calmodulin kinase II (CaMKII) phosphorylation in the dorsolateral quadrant is the highest 5–10 min posttraining (Modarresi et al. 2016). An antagonist of CaMKII prevents the learning of a specific peppermint preference (Modarresi et al. 2016) suggesting odor preference specificity requires glutamatergic recruitment of this mediator. While GluN1 NMDARs have returned to baseline by 24 h, the NMDAR 2B subunits are significantly lower than baseline at 24 h (Lethbridge et al. 2012), implying that the odor learning circuit is less plastic, and more stable, at the time of 24 h memory expression.

Consistent with more robust and stable odor activation, the AMPAR GluA1 subunits are significantly increased at 24 h as measured in synaptosomes (Cui et al. 2011). The increase in AMPAR GluA1 subunits is selective to synaptic insertion as there is no change in total tissue GluA1. Using immunohistochemistry (IHC) Cui et al. (2011) demonstrated increased dorsolateral glomerular GluA1 expression at 24 h but not 48 h. Critically, preventing AMPA receptor insertion blocked 24 h early odor preference memory (Cui et al. 2011).

Correspondingly, in ex vivo experiments, the olfactory nerve evoked-mitral cell AMPAR currents are increased at 24 h, while NMDAR currents are decreased (an increased AMPAR/NMDAR current ratio) (Lethbridge et al. 2012). Similar effects are seen in the occluded nares lateralized odor preference model (Fontaine et al. 2013). These functional outcomes are consistent with postsynaptic AMPAR insertion in mitral cell glomerular dendrites mediating long-term potentiation-like changes in the olfactory nerve to mitral cell synapses underlying peppermint representations. Additionally, metabolic activity as indexed by intrinsic light reflection is increased at 24 h over the surface of the dorsal OB of trained rat pups at the glomerular level (Yuan et al. 2002). Thus, increased metabolic support also occurs in the glomeruli following a single training trial.

Arc *cat*FISH methodology, which enables a comparative visualization of peppermint odor representations before and after training, shows that both excitatory mitral cell and inhibitory granule cell representations for peppermint in the dorsolateral quadrant become significantly more stable with training (Shakhawat et al. 2014). There is greater overlap in both the excitatory and the inhibitory representations indexed by in situ Arc expression in mitral and granule cells (Shakhawat et al. 2014).

Here we use LMD of an OB area (see Fig. 1) that contains peppermint-responsive mitral cells (McLean et al. 1999) and microarray analysis of mRNA expression near the end of the 1-h protein synthesis-sensitive period (Grimes et al. 2011) to identify putative mitral cell mRNA mediators of 24 h peppermint odor memory. We examined training-related mRNA transcription occurring near the end of the first hour posttraining (50 min posttraining) at a time when protein synthesis inhibitors prevent intermediate and long-term memory expression while allowing maximal time for training-related transcription events to have been initiated (Fig. 1).

**Figure 1.**
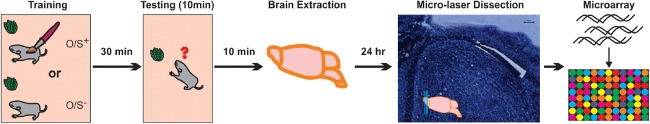
Timeline for peppermint odor or odor + stroking training, odor preference testing and LMD followed by microarray analysis. The thionin-stained section shows a region of the mitral cell layer (MCL) (excised) that was microdissected prior to sample collection in an assay tube. The bar indicates 100 microns. The lasered region is in a dorsolateral quadrant of the MCL normally activated by peppermint.

The acquisition of an odor preference, or its absence, was confirmed prior to sacrifice in each pup. While testing may initiate some transcription, short-term memory is not related to protein synthesis, and it was important for this first study to ensure appropriate behavioral expression of odor preference in both control and experimental pups. Later, in separate groups of rat pups, immunohistochemistry was used to visualize the MCL protein synthesis changes predicted by a subset of the protein-encoding mRNA changes discovered. Whole bulb changes were also examined in trained versus odor only rat pups, using microarrays, to assess the importance of the targeted LMD of mitral cells for the detection of training-related transcription differences in the topographically organized OB.

## Results

### Olfactory testing results

**Figure 2.**
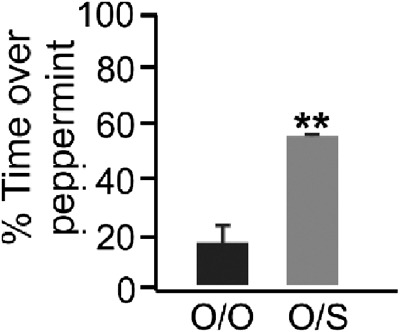
Preference for peppermint after 10 min training session. Black bar represents pups exposed to peppermint odor only during training. Gray hatched bar represents pups given intermittent stroking while exposed to peppermint odor. Unpaired *t*_(4)_ = 4.60, **P < 0.005. Error bars represent standard errors of the mean (SEMs).

Trained pups that had a 50% or better preference score in testing 30 min after training were used for microarray analyses, while odor only exposed pups were selected that showed 30% or less preference at test. Group behavioral performance is shown in Figure 2. Early odor preference learning for peppermint following the odor + stroking condition has been reflected in a reduction in avoidance of peppermint in week-old rat pups since it was first reported (22% odor only versus 60% odor + stroking Sullivan and Leon 1987).

### Laser-dissected dorsolateral mitral cell layer microarray

Twenty-four genes had a significantly different expression in the laser-dissected dorsal lateral MCL of the OB in pups that learned an odor preference for peppermint versus control pups that were only given odor exposure and did not develop a preference. Of these, 13 were nonprotein-coding genes (see Table 1) and eleven were protein-coding genes (see Table 2). (Supplemental Spreadsheet 1 provides all the microarray data for the LMD experiment.)

**Table 1.**
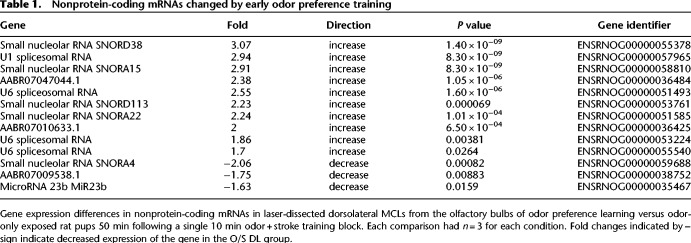
Nonprotein-coding mRNAs changed by early odor preference training

**Table 2.**
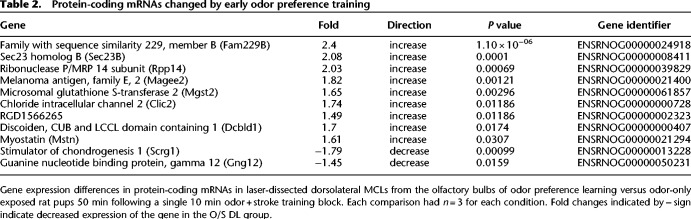
Protein-coding mRNAs changed by early odor preference training

### Nonprotein-coding mRNAs (*n* = 13)

Five small nucleolar RNAs, involved in the maturation of noncoding mRNAs (Smalheiser 2014) differed by microarray analysis. Four, Snord38, Snord113, Snora15, and Snora22, were elevated in the learning group, while Snora4 was decreased. Four small nuclear RNAs, U1 (1) and U6s (3), which are components of spliceosomes, were also elevated in the learning group. Finally, four microRNAs were significantly changed with training. Three are novel microRNA precursors about which little is known. Two were up-regulated, AABR07010633.1 and AABR0704047044, while AABR07012826.1 was down-regulated. The fourth, a well-characterized microRNA, MiR23b, was down-regulated with training.

Noncoding mRNAs modulate dendritic protein translation and are altered in the hippocampus in the early stages of two-odor discrimination learning in adult rats (Smalheiser 2014). Modulating local translation of dendritic protein synthesis can permit rapid alteration of synaptic connectivity and provide dynamic, time-limited support of changing functional connectivity.

Spliceosome changes relate to splicing variations in proteins and the expression of differing isoforms. Splicing permits alteration of functional proteins without changing the levels of protein synthesis per se. The microarray used identified protein isoforms only fortuitously. But, alterations in the gene probes of one protein-encoding mRNA (Sema4C, see Immunohistochemistry results) were consistent with a splicing change as part of 24 h odor preference learning.

**Figure 3.**
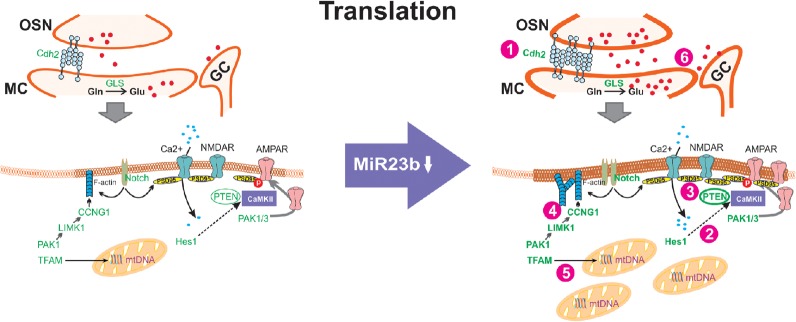
Model of functional alterations associated with the protein translation increases predicted by down-regulating MiR23b in the dorsolateral mitral cells of the olfactory bulb in learning versus nonlearning pups. Genes highlighted in green are also in mitral cells in mice in this age range (Developing Mouse Brain Atlas). Functional changes include (1) increases in cell adhesion, (2) increases in AMPA receptors, (3) modulation of NMDA receptors, (4) increases in the synaptic membrane and actin branching, (5) increases in mitochondrial biogenesis, (6) increases in neuronal glutamate synthesis. (OSN) olfactory sensory nerve, (MC) mitral cell dendrite, (GC) granule cell inhibitory interneuron dendrite, (Cdh2) cadherin2 or N-cadherin, (Gln) glutamine, (GL) glutamate, (GLS) glutaminase, (Ca^2+^) calcium, (NMDAR) NMDA receptor, (AMPAR) AMPA receptor, (mtDNA) mitochondrial DNA, (TFAM) Transcription Factor A Mitochondrial, (LIMK1) Lim-domain kinase, (CCNG1) cyclin G1, (F-actin) filamentous actin, (Notch) Notch Receptor, (PSD95) postsynaptic density protein 95, (Hes1) Hairy enhancer of split protein1, (PTEN) Phosphatase and tensin homolog deleted on chromosome 10, (PAK1 and PAK1/3) Serine/Theronine-activated p21 kinases, (CaMKII) calmodulin kinase II, (P) phosphate.

MicroRNAs (MiR) have a direct role in modulating protein translation. Their predominant role is the inhibition of translation of specific mRNA targets. Thus, down-regulation of MiRs can rapidly up-regulate translation of proteins supporting circuitry change. More than 20 mRNAs implicated in synaptic plasticity have been validated for MiR23b (see Discussion), while all were expressed in the microarray samples, the Translation model proposed in Figure 3 is confined to nine plasticity-related mRNAs (Cdh2, Gls, Tfam, Pak1, Limk1, Ccng1, Notch, PTEN, Pak1, and Hes1) confirmed to be expressed in mitral cells of the mouse dorsolateral OB at PND4 and PND14 as documented in the Allen Developing Mouse Brain Atlas (2008). Their functional roles in synaptic plasticity support are identified in Figure 3. While in situ hybridization identifies their production in mitral cells, translation is also likely to occur locally at synaptic sites, but levels of mRNA expression at those sites do not permit visualization in the Allen Developing Mouse Brain Atlas (2008). Glomerular expression is visible for Cdh2, Gls, Pak1, LimK1, Ccng1, Notch2, Pten, and Pak1 in this age range, but may relate to expression in juxtaglomerular neurons rather than mitral cell glomerular dendrites.

### Protein-coding gene changes (*n* = 11)

Nine protein-coding mRNAs were up-regulated: Sec23b, Clic2, Rpp14, Mstn, Dcbld1, Magee2, Fam229b, RGD1566265, and Mgst2 (see Table 1). Four of these were assessed for optical density changes in protein expression using IHC in the occluded naris learning model: Sec23b, Clic2, Magee2, and Dcbld1. In the occluded naris model rat pups have one naris occluded during the 10 min odor + stroking training session. The bulbs were removed for IHC analysis at the same time point (50 min posttraining) as for the microarray experiments. Two protein-coding mRNAs were down-regulated: Scrg1 and Gng12. Scrg1 was also assessed by IHC in the occluded naris model. Since an earlier Sick Kids (University of Toronto) microarray analysis, which separated gene probes rather than treating all gene probes for one protein mRNA species as a single index of production of that protein as done here, suggested the Sema4c expression was regulated by training, we also used an available Sema4C antibody for IHC. IHC confirmed mitral cell localization of Sema4c and demonstrated up-regulation for the isoform detected by the antibody.

All of the protein-coding mRNAs modified by training in the 6-d old OB were confirmed to be actively expressed in mitral cells of the PND56 mouse brain in the Allen Brain Atlas (Lein et al. 2007), but only Mstn (myostatin) was displayed in the Allen Developing Mouse Brain Atlas (2008). Mstn is expressed in mitral cells at both PND4 and PND14. Mtsn is best known for its role in negatively regulating muscle cell growth, but myostatin protein and the myostatin propeptide occur in rodent mitral cells (Iwasaki et al. 2013). Specifically, in adult mouse, myostatin is selectively expressed in mitral cells implicated in mediating learned odor aversion (Iwasaki et al. 2013). The present microarray result suggests Mstn also plays a role in those mitral cells mediating early learned odor preference.

**Figure 4.**
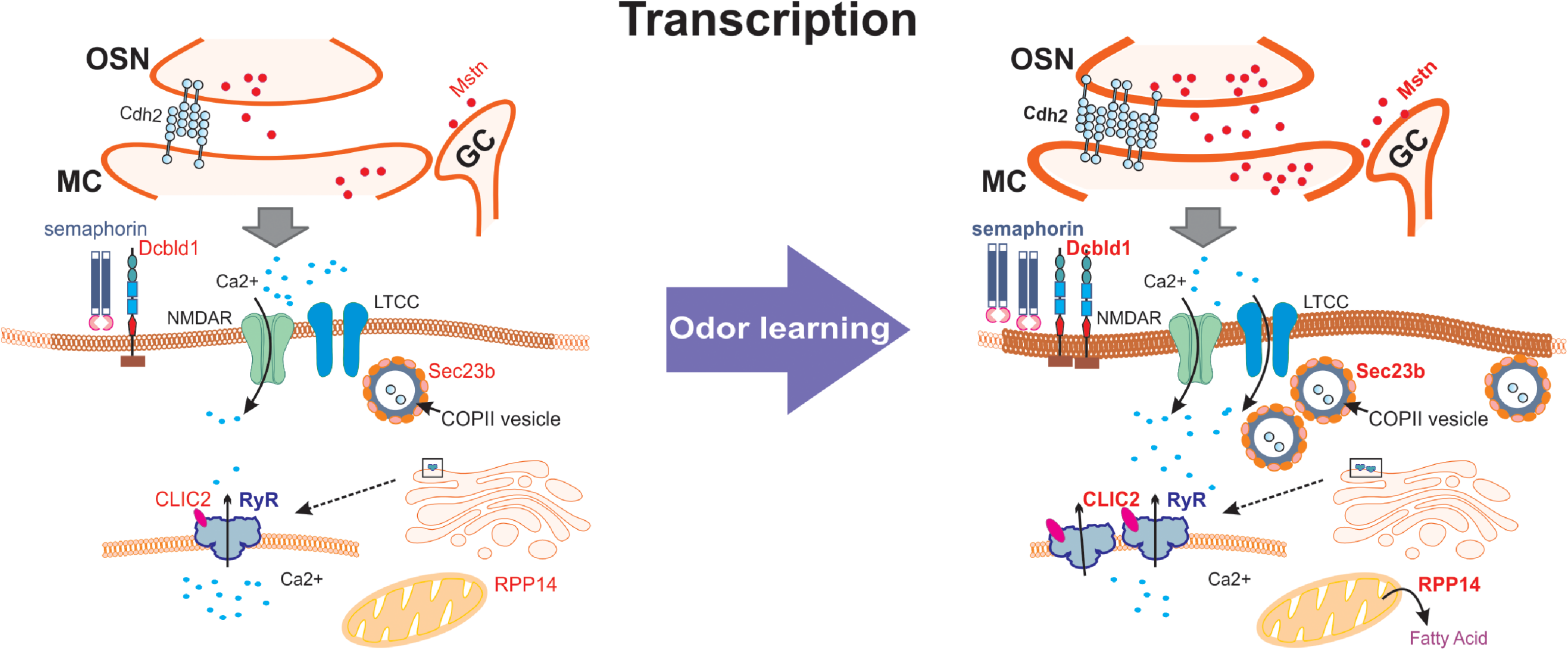
Model of training-induced plasticity changes predicted by up-regulation of transcription in five functionally characterized proteins in the microarray. The protein-coding genes in red support structural changes such as increased connectivity (Dcbdl1, Sec23b), increased synaptic membrane (Sec23b), increased ryanodine receptor clustering (Clic2), increased fatty acid production for mitochondrial biogenesis (Rpp14), and increased excitatory input to granule cells (Mstn).

A model of the functional effects supported by the changes in protein-encoding mRNAs that have been well-characterized is shown in Figure 4.

### Immunohistochemistry demonstrating protein expression in the rat pup occluded naris model in a subset of protein-coding genes identified as learning-related by microarray

It does not appear that any of the proteins we examined with IHC has been previously demonstrated in mitral cells of the rat pup OB. All were present in mitral cells.

Dcbld1 belongs to a novel class of two transmembrane scaffolding proteins that are structurally similar to the neuropilins, containing a discoiden domain (DC) and a complement C1r/C1s/Uegf, Bmp1 (CUB) domain. The CUB domain provides a site for semaphorin interaction (Schmoker et al. 2019). CUB domains can also interact with glutamate receptors, e.g., (Ng et al. 2009). Generally, neuropilins are receptors for plexins or semaphorins and guide the development of neuronal connections. While initially linked to axonal guidance (Gavazzi 2001), they are now known to play critical roles in dendritic guidance (Kim and Chiba 2004). Semaphorin4B targets Dcbld2 for ubiquitylation (Schmoker et al. 2019). Schmoker (Schmoker et al. 2019) provides an excellent review of what is known about Dcbld proteins.

**Figure 5.**
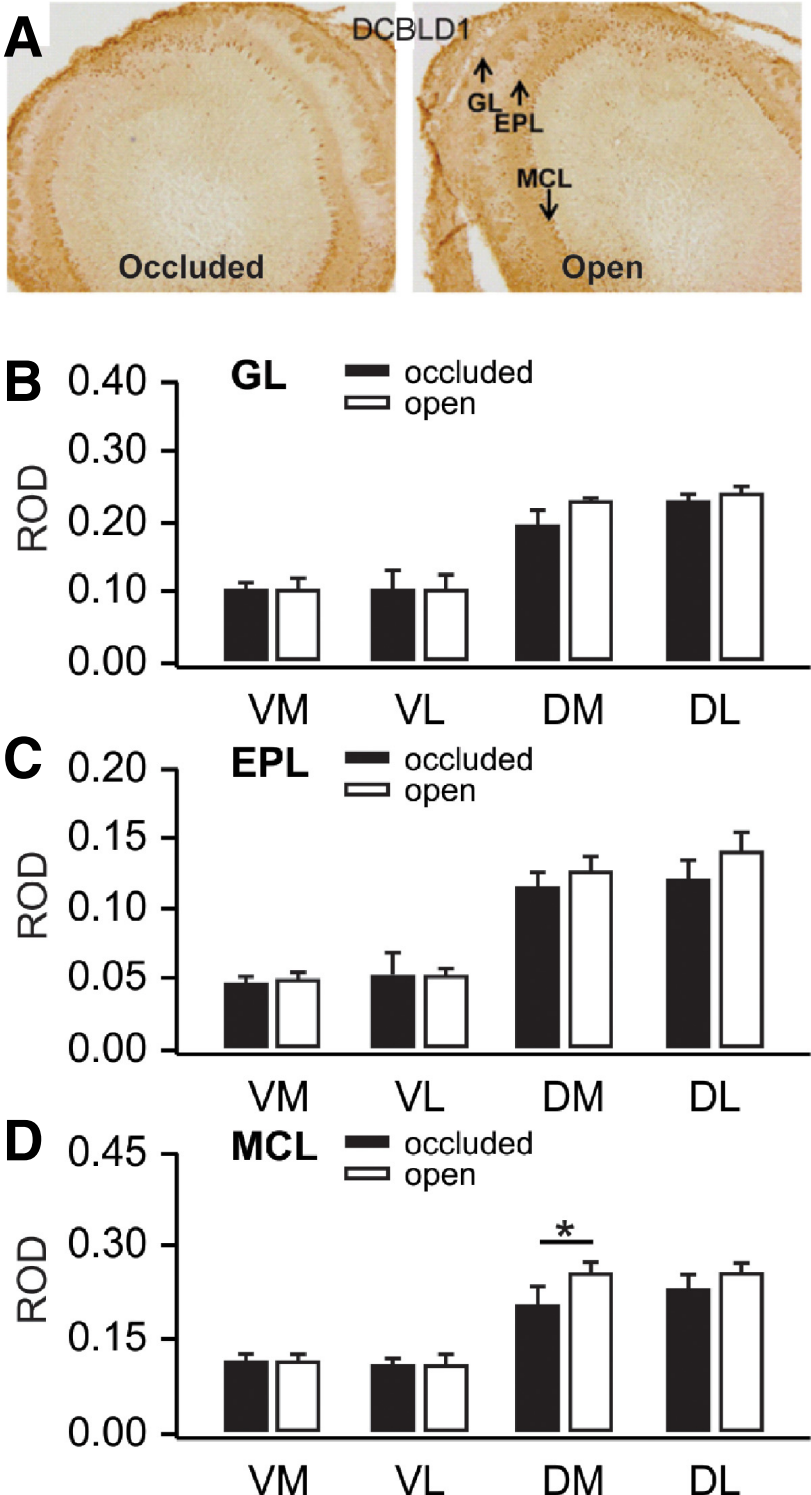
(*A*) Dcbld1 immunohistochemical staining in olfactory bulbs ipsilateral to open and closed nares 50 min following odor stroking training. (*B*) Glomerular layer ROD values for olfactory bulb quadrants receiving input from open and closed nares. (*C*) External plexiform ROD values for olfactory bulb quadrants receiving input from open and closed nares. (*D*) Mitral cell layer ROD values for olfactory bulb quadrants receiving input from open and closed nares. Error bars represent SEMs. * *P* < 0.05, see results for exact values.

Dcbld1 protein was present in the MCL, the external plexiform layer (EPL) and the glomerular layer (GL) of the OB (Fig. 5A). Hemisphere (occluded or open nares) and quadrants (ventral medial, VM, ventral lateral, VL, dorsal medial, DM, dorsal lateral, DL) were assessed for differences in IHC optical density. ANOVAs were not significant for Hemisphere or Hemisphere × Quadrant interactions for EPL (Hemisphere *F*_(1,14)_ = 1.892, *P* = 0.191, Hemisphere × Quadrant *F*_(3,14)_ = 0.720, *P* = 0.556) or GL (Hemisphere *F*_(1,14)_ = 3.848, *P* = 0.07; *F*_(3,14)_ = 2.053, *P* = 0.153). However, in MCL, Hemispheres were significantly different, *F*_(1,14)_ = 5.851, *P* = 0.030, as were Quadrants, *F*_(3,14)_ = 17.677, *P* < 0.001, although Hemisphere × Quadrant (*F*_(3,14)_ = 2.513, *P* = 0.101) was not significantly different. See Figure 5B–D. A post-hoc paired *t*-test revealed darker reactivity in the dorsal medial portion of the MCL in the open nares hemisphere, *t*_(4)_ = 2.971, *P* = 0.041.

**Figure 6.**
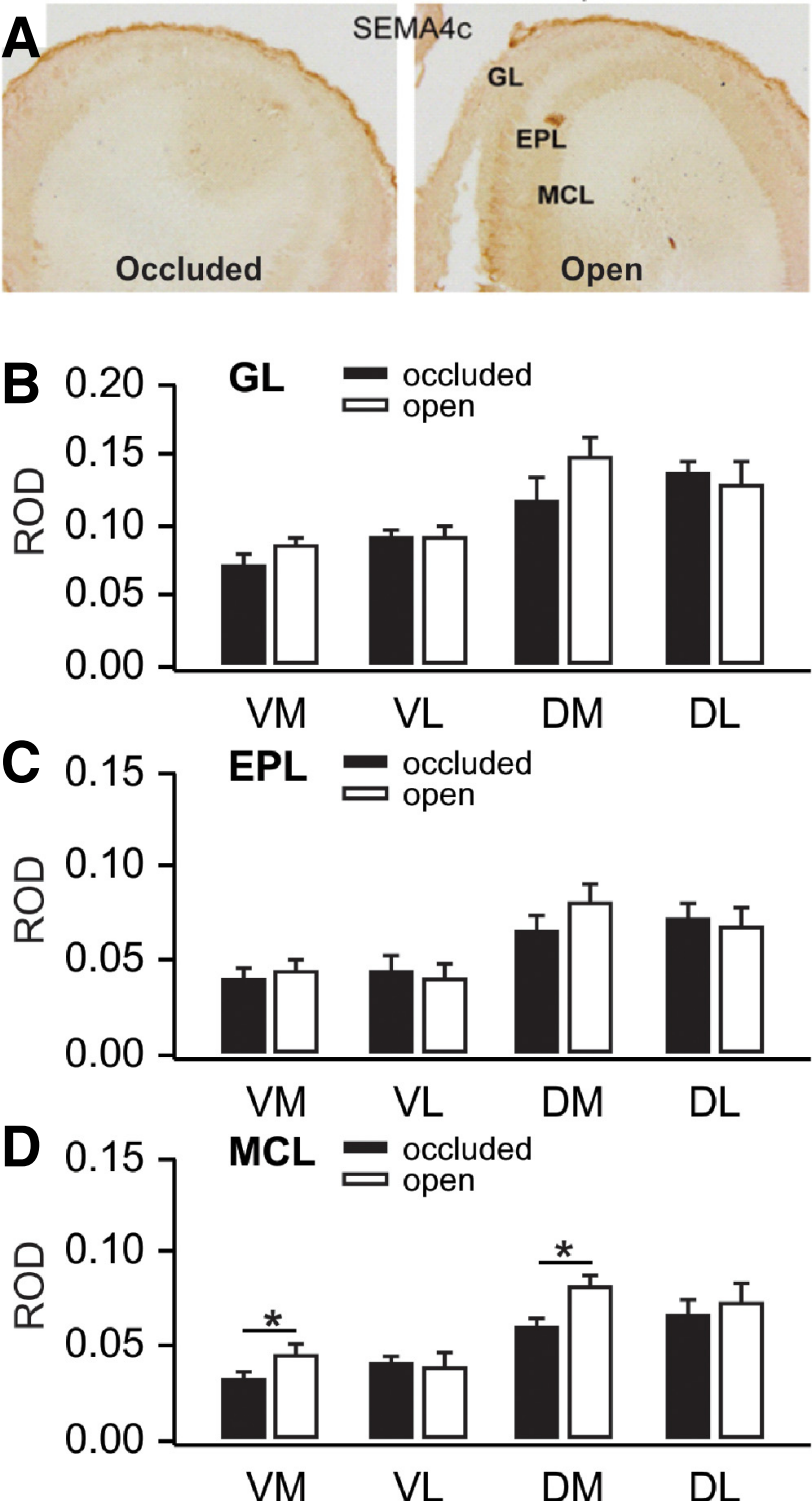
(*A*) Sema4c immunohistochemical staining in olfactory bulbs ipsilateral to occluded and open nares 50 min following odor + stroking training. (*B*) Glomerular layer ROD values for olfactory bulb quadrants receiving input from occluded and open nares. (*C*) External plexiform ROD values for olfactory bulb quadrants receiving input from occluded and open nares. (*D*) Mitral cell layer ROD values for olfactory bulb quadrants receiving input from occluded and open nares. Error bars represent SEMs. * *P* < 0.05, see results for exact values.

As mentioned earlier, we also examined Sema4C with IHC (Fig. 6A). In an initial analysis of our microarray data, for which each gene-related probe was treated separately, a semaphorin, identified as Semaphorin 4C, was down-regulated by odor preference training. We tested a Sema4C antibody in IHC and found up-regulation by training. The difference in outcome appears to lie in splicing variants. In the current analysis, gene-probes identifying the same protein were grouped together as a single index of that protein. The multiple probes for Semaphorin 4c, together, were not significantly different after training. One Semaphorin 4c splicing variant (Semaphorin4c-201) was significantly down-regulated as initially identified, but when combined with the probe levels of another Semaphorin 4c variant (Sempaphorin4c-001), the reduction in Semaphorin 4c overall disappeared. Semaphorin4c-001 mRNA increased and the increase in Semaphorin4c-001 was sufficient to cancel out the previously significant Semaphorin4C-201 decrease.

In the IHC, using a commercial Sema4C antibody whose variant specificity is unknown, we found higher optical density for Sema4C on the open nares side with training (see Fig. 6B–D) consistent with the Dcbld1 data above. In the Hemisphere and Hemisphere × Quadrant ANOVAs there were no differences for the GL (Hemisphere *F*_(1,14)_ = 2.79, *P* = 0.117, Hemisphere × Quadrant *F*_(3,14)_ = 2.511, *P* = 0.101 or the EPL (Hemisphere *F*_(1,14)_ = 1.44, *P* = 0.25, Hemisphere × Quadrant *F*_(3,14)_ = 2.416, *P* = 0.110) regions, but the MCL had a significant Hemisphere effect, *F*_(1,14)_ = 9.38, *P* = 0.008, and a significant Quadrant effect, *F*_(3,14)_ = 12.575, *P* < 0.001, although again the Hemisphere × Quadrant effects was not significant (*F*_(3,14)_ = 1.745, *P* = 0.204). Sema4C was significantly darker for the open nares hemisphere in the ventral medial (*t*_(3)_ = 4.661, *P* < 0.010) and dorsal medial quadrants (*t*_(3)_ = 4.49, *P* = 0.021). The dorsal pattern of this Sema4C antibody (Fig. 6A) aligned with that of the Dcbld1 antibody (Fig. 5A), the neuropilin-like possible partner for semaphorins in generating neuronal connectivity.

Clic2 acts primarily as a modulator of the ryanodine receptors controlling intracellular calcium release from the endoplasmic reticulum by stabilizing its closed state and enhancing substate events (Dulhunty et al. 2005; Meng et al. 2009). Clic2 effects on the ryanodine receptor resemble those produced by the calcineurin inhibitor FK506 (Brillantes et al. 1994), which acts in concert with one-trial training to extend early odor preference memory (Christie-Fougere et al. 2009).

**Figure 7.**
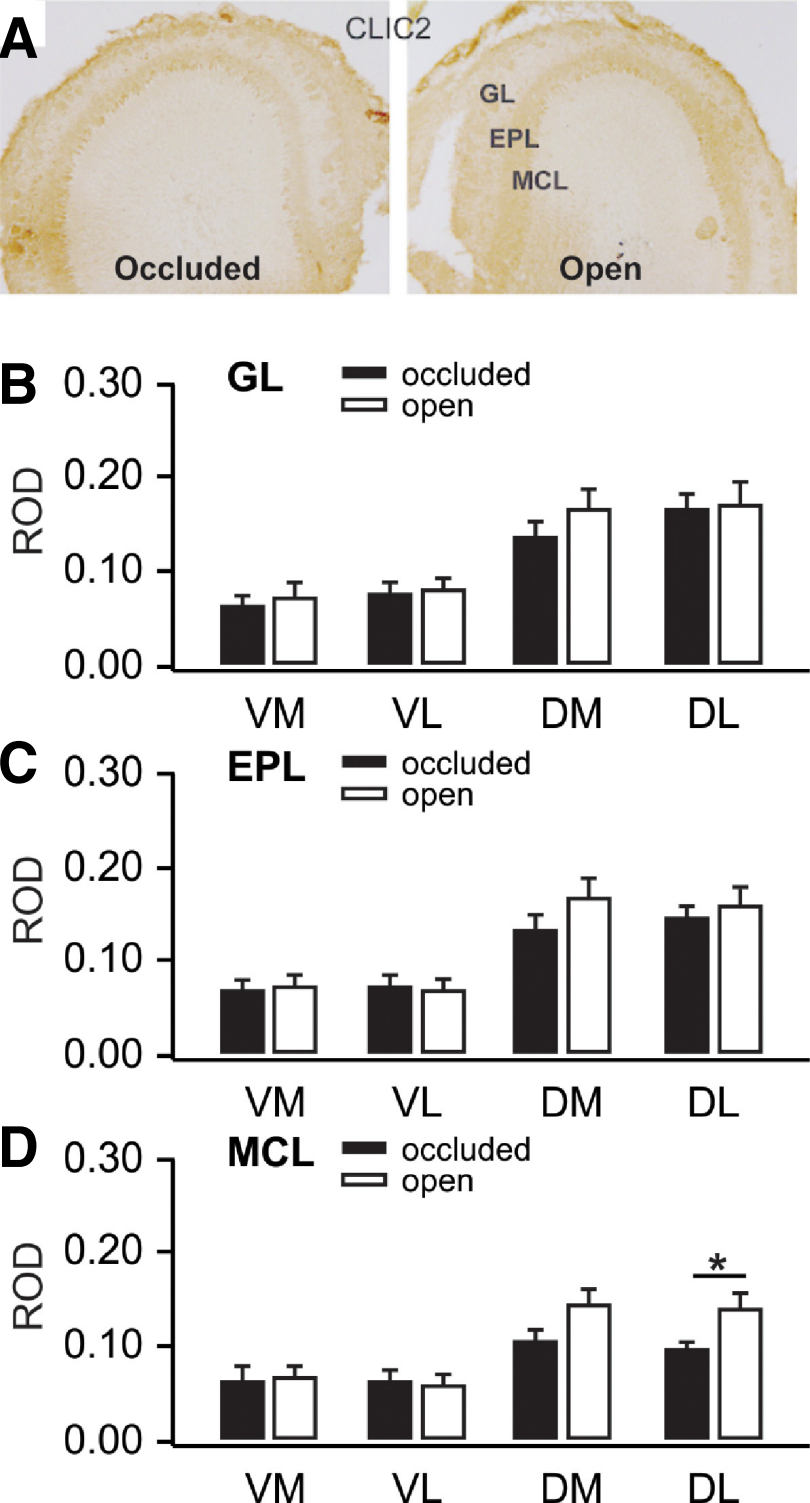
(*A*) Clic2 immunohistochemical staining in olfactory bulbs ipsilateral to occluded and open nares 50 min following odor + stroking training. (*B*) Glomerular layer ROD values for olfactory bulb quadrants receiving input from occluded and open nares. (*C*) External plexiform ROD values for olfactory bulb quadrants receiving input from occluded and open nares. (*D*) Mitral cell layer ROD values for olfactory bulb quadrants receiving input from occluded and open nares. Error bars represent SEMs. * *P* < 0.05, see results for exact values.

In the unilateral nares experiment, IHC revealed that Clic2 protein was strongly expressed in mitral cells as well as in the glomerular and external plexiform regions (see Fig. 7A). ANOVAs for Hemisphere and Hemisphere × Quadrant were not significant for the GL (Hemisphere *F*_(1,18)_ = 4.799, *P* = 0.042, Hemisphere × Quadrant *F*_(3,18)_ = 1.433, *P* = 0.266) or EPL (Hemisphere *F*_(1,18)_ = 2.430, *P* = 0.136, Hemisphere × Quadrant *F*_(3,18)_ = 1.592, *P* = 0.226), but were significant for MCL: Hemisphere, *F*_(1,18)_ = 9.542, *P* = 0.006, Hemisphere × Quadrant, *F*_(3,18)_ = 3.597, *P* = 0.034. See Figure 7B–D. Paired *t*-tests revealed that dorsal lateral mitral cells were significantly more reactive in the open nares hemisphere, *t*_(5)_ = 2.758, *P* = 0.040.

Scrg1 mRNA produces a small secreted-peptide up-regulated in transmissible spongiform encephalopathies (Dron et al. 1998). Its neuronal expression increases postnatally in mice, reaching adult levels by PND8 (Dron et al. 2000). Scrg1 also marks dense core vesicles (Dandoy-Dron et al. 2003). Scrg1 reduction is associated with differentiation (Aomatsu et al. 2014), while increases can relate to autophagy (Dron et al. 2005).

**Figure 8.**
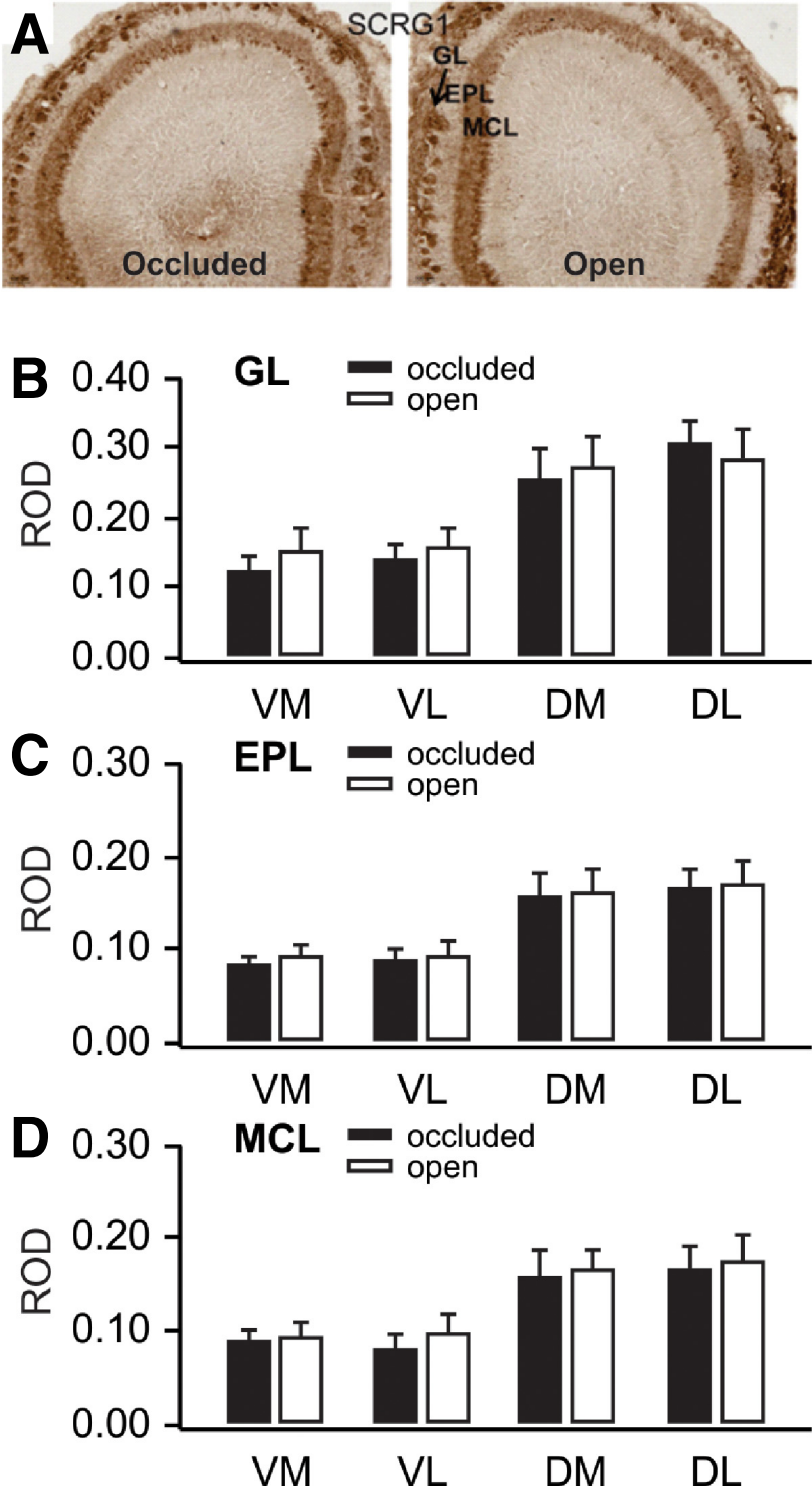
(*A*) Scrg1 immunohistochemical staining in olfactory bulbs ipsilateral to occluded and open nares 50 min following odor + stroking training. (*B*) Glomerular layer ROD values for olfactory bulb quadrants receiving input from occluded and open nares. (*C*) External plexiform ROD values for olfactory bulb quadrants receiving input from occluded and open nares. (*D*) Mitral cell layer ROD values for olfactory bulb quadrants receiving input from occluded and open nares. Error bars represent SEMs.

The IHC results confirmed that SCRG1 protein is targeted to mitral cells and to the EPL and glomerular layer of PND6 rat OB. See Figure 8A. Neither the Hemisphere nor the Hemisphere × Quadrant ANOVAs showed significant differences in SCRG1 reactivity in MCL (Hemisphere *F*_(1,14)_ = 2.392, *P* = 0.144, Hemisphere × Quadrant *F*_(3,14)_ = 3.52 × 10^−5^, *P* = 0.994), EPL (Hemisphere *F*_(1,14)_ = 2.056, *P* = 0.174, Hemisphere × Quadrant *F*_(3,14)_ = 0.026, *P* = 0.994) or GL (Hemisphere *F*_(1,14)_ = 2.834, *P* = 0.114, Hemisphere × Quadrant *F*_(3,14)_ = 3.253, *P* = 0.054). See Figure 8B–D.

Sec23B is a protein component of COPII, a coated vesicle complex for vesicular export (see (McCaughey and Stephens 2018) for recent review). Sec23A/B paralogues appear interchangeable (Khoriaty et al. 2018). In neurons, Sec23 marks endoplasmic reticulum exit sites that are seen at dendritic branch points (Cui-Wang et al. 2012) and at sites of dendritic glutamate receptor insertion (Evans et al. 2017; Pick et al. 2017). Sec23b, itself, has also been implicated in cadherin export (Zhu et al. 2015).

**Figure 9.**
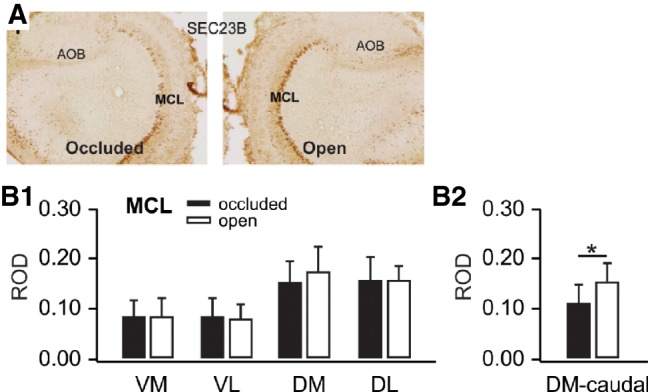
(*A*) Sec23b immunohistochemical staining in olfactory bulbs ipsilateral to occluded and open nares 50 min following odor + stroking training. (*B*1) Mitral cell layer ROD values for olfactory bulb quadrants receiving input from occluded and open nares. (*B*2) Dorsomedial mitral cell differences observed in the caudal samples for Sec23b. Error bars represent SEMs. * *P* < 0.05, see results for exact values.

IHC revealed that Sec23B expression was strongest in mitral cells (see MCL Fig. 9A). There were no significant differences in ANOVAs for Hemisphere or Hemisphere × Quadrant interactions (Fig. 9B1) in any layer except the MCL (GL Hemisphere *F*_(1,14)_ = 2.79, *P* = 0.117, Hemisphere × Quadrant *F*_(3,14)_ = 2.511, *P* = 0.101; EPL Hemisphere *F*_(1,14)_ = 1.44, *P* = 0.25, Hemisphere × Quadrant *F*_(3,14)_ = 2.416, *P* = 0.110; MCL Hemisphere *F*_(1,14)_ = 9.38, *P* = 0.008, Hemisphere × Quadrant *F*_(3,14)_ = 1.745, *P* = 0.204). A paired *t*-test of differences observed in the *cauda*l dorsal MCL revealed stronger reactivity in the open nares hemisphere (Fig. 9B2, *t*_(5)_ = 3.419, *P* = 0.019; see also exemplar sections in Fig. 9A showing caudal dorsal MCL). All error bars represent SEMs.

Magee2 is a member of the Mage family Group II. Members of Mage family Group II are expressed in normal tissues rather than cancers (De Donato et al. 2017). Through the MAGE homology domain, MAGE proteins of Groups I and II interact with RING proteins to promote ubiquitylation that targets substrates for degradation (Doyle et al. 2010). Another member of the MAGE family, necdin, promotes mitochondrial health and biogenesis in mammalian neurons (Hasegawa et al. 2016).

The present study is the first to identify a role for Magee2 in neural plasticity. IHC data confirmed the selective distribution of Magee2 protein in mitral cells, in the EPL and in the glomerular layer of the 6-d old OB (See Supplemental Fig. 1). There was no significant up-regulation of Magee2 protein on the open nares side at 50 min posttraining in the dorsal bulb. Ventral areas were not analyzed.

Since the mRNA differences found in the dorsal LMD region in the microarray at 50 min were not evident in changes in protein expression for SCRG1 or Magee2, at the same time point, the changes in mRNA for these proteins may occur relatively late in the 1 h transcription window. A delayed protein increase in Magee2 might support AMPA receptor degradation and the termination of memory expression after 24 h. See Widagdo et al. (2017) for an example. The role of a delayed decrease in SCRG1 is less clear, although it could link to increasing differentiation in the learning circuit.

One caveat in considering the evidence for higher expression of proteins in dorsally placed mitral cells on the open nares side in these experiments is that preventing odor input by a 10 min nares occlusion on the closed side may, itself, reduce OB protein expression. However, elevated protein expression on the side of the open nares was not seen for either Srcg1 or Magee2 suggesting it is not a general functional effect. Additionally, previous work with the occluded nares paradigm in our laboratory did not find differences between open and closed nares for the protein calmodulin kinase II with training in PND6 rat pups, although phosphorylated calmodulin kinase II increased on the open nares side (Modarresi et al. 2016).

### Whole bulb microarray results

There were no significant differences in the microarray analyses using trained and untrained whole OBs for rat pups sacrificed 50 min posttraining (Supplemental Spreadsheet 2 provides the data from the whole bulb microarray analysis). This outcome suggests the mRNA changes supporting odor preference learning are selective for the LMD mitral cell sample and are masked in whole bulb analysis.

## Discussion

In this first study of mRNA expression related to 24 h, rather than weeks-long memories, in the mammalian brain, the majority of mRNA increases are in support of enhanced mRNA translation and/or altered mRNA splicing rather than increased or decreased overall protein transcription. The 13 noncoding mRNAs are linked to translation, translation support (Smalheiser 2014), or to splicing. Fortuitously, as microRNAs were not a specific microarray target, the decrease in MiR23b that was revealed highlighted a brain-enriched microRNA biologically validated to down-regulate more than 20 mRNAs related to neural circuit plasticity including MMP9, Plaur/uPA, Psap, Arf, Arhpgef6, Anxa2, Oprm1, Pak3, Bcl-xl, Vldlr, Apaf1, Hip1r, LimK3, Runx2, and Pyk2/Ptk2b. Another 9 mRNAs, Cdh2, Gls, Tfam, Pak1, Limk1, Ccng1, Notch, PTEN, Pak1/3, and Hes1 that are down-regulated by MiR23 have been shown in mitral cells in mice at both PND4 and PND14. In the initial Sick Kids statistical analysis of microarray data mentioned earlier, dorsal OB samples were compared to ventral OB mitral cell samples. While comparative dorsal versus ventral results were hard to interpret and were not pursued in the present analysis, the largest significant difference between the dorsal and ventral LMD samples for the trained rat pups was the down-regulation of Mir23b mRNA in the dorsal mitral cell LMD samples relative to the ventral mitral cell LMD samples, consistent with the between-group down-regulation of Mir23b reported here.

The functional effects predicted by down-regulation of Mir23b that are related to increases in the translation of the nine mRNAs confirmed in neonatal mouse mitral cells are illustrated in Figure 3.

All 20 of the Mir23b-regulated plasticity mRNAs listed are expressed in the dorsolateral mitral cells of the adult mouse as seen in sagittal sections in the Allen Mouse Brain Atlas (Lein et al. 2007) and all were observed in the week-old rat pup microarray here, but only the subset illustrated in Figure 3 was confirmed in neonatal mouse mitral cells (Allan Developing Mouse Brain Atlas). None of the 20 Mir23b targets were transcriptionally altered.

The predicted increase in Cdh2 translation due to down-regulated MiR23b is consistent with the cadherin-mediated model of mammalian learning and memory proposed by Lisman (Sanhueza and Lisman 2013). The Lisman cadherin model specifically relates to learning initiated by NMDA receptors and activated CaMKII, which are both engaged in early odor preference learning (Lethbridge et al. 2012; Modarresi et al. 2016).

MicroRNAs have an unusual temporal profile with rapid production associated with rapid turnover (Li et al. 2013). We are only beginning to understand the temporospatial regulation of these neuronal plasticity controllers (Kiltschewskij and Cairns 2019). Mir23b down-regulation with a single training trial may be temporally limited such that it effectively supports 24 h, but not 48 h, memory. However, MiRs are also implicated in multiday long-term memories (Korneev et al. 2018). It will be of interest to examine differences in mRNA expression in trained and nontrained cohorts at 48 h.

The US for preference memory, a cAMP increase, may down-regulate Mir23b through pCREB recruitment of AP1. AP1 is highly expressed in the first three postnatal weeks (Pennypacker et al. 1995) and its activation suppresses Mir23b (Pellegrino et al. 2013). MiR23b can also be down-regulated by DNA methylation (Majid et al. 2012; Kou et al. 2016) initiated by cAMP increases (Fang et al. 2015).

The importance of noncoding proteins involved in splicing variation also deserves highlighting as the observations here with the two Semaphorin 4C isoform probes illustrates. It will be important in future studies to specifically target the issue of training-induced isoform change.

Of the nine protein-coding mRNAs up-regulated by early odor preference acquisition during the first hour posttraining, four have known functions: Sec23b, Rpp14, Clic2, and Mstn. Sec23b is involved in membrane expansion and vesicular export. Consistent with this role, in Drosophila the homolog of Sec23B is associated with the elaboration of dendrites, but not axons (Ye et al. 2007). An increase in cAMP would activate CREB3, which regulates transcription of COPII components like Sec23b (Iyer et al. 2013). CREB3 was expressed at high levels in the LMD samples (see Supplemental Spreadsheet 1).

Rpp14 mRNA encodes a subunit of the multiprotein RNA complex, Ribonuclease P. Rpp14 with the support of opa-interacting protein 2, acts as an exonuclease removing the 3′ end of tRNAs (Jiang and Altman 2002) to generate tRNA fragments. tRNA fragments have been associated with synaptic vesicles and hypothesized to promote local protein synthesis (Li et al. 2015). Rpp14 is also a bicistronic mRNA. The second protein made, mitochondrial 3-hydroxyacyl thioester dehydratase, stimulates mitochondrial fatty acid synthesis (Autio et al. 2008) and would support mitochondrial biogenesis (Fig. 4) to enhance energetic/metabolic support of learning circuitry.

Increases in Clic2 mRNA index a change in intracellular calcium regulation. Clic2 stabilizes the closed state of ryanodine receptors (Meng et al. 2009), acting similarly on those receptors to the exogenous agent FK506 (Brillantes et al. 1994) as mentioned. FK506 increases the strength and duration of early odor preference memory (Christie-Fougere et al. 2009) consistent with a role for ryanodine receptors in early odor preference learning. Clic2 increases would be predicted to support odor learning based on the FK506 findings. The highest density of ryanodine receptors occurs in the EPL of the OB (Padua et al. 1992). Ryanodine clustering promoted by NMDAR-related calcium entry has been hypothesized to support synaptic plasticity in the postnatal period (Lee et al. 2016). β-receptor activation also acts to enhance calcium sparks triggered from the ryanodine receptor in response to calcium entry through single L+ voltage-gated calcium channels (Zhou et al. 2009) that, themselves, have a pivotal role in early olfactory preference learning (Jerome et al. 2012).

Myostatin, best known for its role in regulating muscle growth, appears here to have a role in modulating mitral cell circuitry to support odor preference learning. Earlier experiments have demonstrated bulbar mitral cell increases in myostatin in mouse odor aversion learning (Iwasaki et al. 2013). Extracellular OB myostatin release also activates c-fos in granule cells (Iwasaki et al. 2013). Increased activation at the mitral/granule cell synapse is promoted by OB NE input in rat pups less than 14 d old and is associated with long-term increases in OB gamma oscillations (Pandipati and Schoppa 2012). The consistent participation of specific mitral/granule cell circuitries in early odor learning is evidenced by increased stability of arc signatures in both cell types in the dorsolateral OB after early odor preference training (Shakhawat et al. 2014).

Myostatin gene expression is up-regulated by CREB activation (Zuloaga et al. 2013), the initiator of early odor preference learning. Some studies suggest myostatin might decrease synaptic strength (Augustin et al. 2017). It is likely that both increased and decreased synaptic changes occur with learning to reflect new input control of behavior and to maintain synaptic homeostasis, respectively. Its mechanistic role here will be of interest.

The remaining proteins-encoding mRNAs have not been characterized functionally, five were up-regulated: Fam229b had the largest fold change with training; RGD1566265 is located on the X chromosome; Mgst2 catalyzes production of the C4 leukotriene (Ahmad et al. 2015); Magee2 and Dcbld1 may both be predicted to have roles in new neuronal connections, but definitive studies are not available. The down-regulated protein Gng12, the G protein gamma subunit12, is associated with filamentous actin and observed in both glia and postsynaptic neuronal compartments. The other down-regulated protein, Srcg1, is a scrapie-related protein targeted to neuronal dense-core vesicles and associated with autophagy in injury models.

Taken together the mRNA changes with known functions suggest that 24 h memory is supported primarily by increased translation, possibly local, of proteins that alter the strength of functional communication for the glutamatergic olfactory nerve input to mitral cells and the glutamatergic mitral cell output to granule cells. Congruent with these dynamic functional changes are changes in structural adhesion and the extent of the synaptic membrane. Mitochondrial resources are also promoted in mitral cells mediating 24 h memory.

Structural and energetic changes are also supported by protein transcription changes and would be likely to be more enduring than the functional changes engaged by translation-dependent effects. Neuroarchitectural changes would provide a substrate for the construction of longer-term dynamic memory support with repeated spaced training. A distinction between transient functional changes and more enduring structural changes with LTP has recently been proposed in another model plasticity system (Wiegert et al. 2018).

The week-old rat pup OB offers a highly tractable model for illuminating the biology of mammalian learning and memory. Critical issues in memory support including local translation (Rangaraju et al. 2017) and temporal dynamics (Kukushkin and Carew 2017) will lend themselves to study in this model. Multiple other control conditions will be of interest. Here we chose “odor only” pups as controls since neither odor only or stroking only produces pCREB increases in mitral cells (McLean et al. 1999), but the novel peppermint odor would have activated the mitral cells being dissected. Naive pups, pups with stroking only, pups that were subject to training procedures, but did not show behavioral evidence of having learned, and pups given a single odor + stroking trial and a pharmacological manipulation to induce multiple-day memory (Bhattacharya et al. 2017) as well as those given training parameters that engage only short-term or intermediate-term memory, which don't depend on transcription, (Grimes et al. 2011) will be important comparisons for a fuller understanding the roles of the changes described here.

The novel findings of the present microarray evaluation of learning versus nonlearning pups can readily be probed using in situ and IHC methods to visualize the predicted mRNA and protein changes at varying time points and locations. Bulbar pharmacological as well as oligosense and antisense nucleotide manipulations will permit assessment of the causal roles of identified proteins and mRNAs. Kandel's cAMP/PKA/CREB memory model also predicts CPEB up-regulation when longer-term memories are initiated (Kandel 2012). That can be tested in future studies.

## Materials and Methods

### Animals

Postnatal day (PND) six Sprague Dawley (SD) rats were used for the study, with the day of birth considered PND0. The study was approved by the Institutional Animal Care Committee of Memorial University of Newfoundland. The litters were culled on PND1 to a maximum of 12 pups per litter, with no more than one male and one female pup per litter for each condition (odor only and odor + stroking) used for the study. The rats were on a reverse light–dark cycle (12 h each, lights off at 12 noon) and had access to food and water ad libitum. Twelve rat pups were used to generate the results presented.

### Behavior: training and testing

On PND6, the dam and litter were brought to the behavior room in their home cage. Pups were labeled and then placed back into the home cage 10 min before the training started.

Peppermint scented bedding was prepared in a fume hood by mixing 0.3 mL of peppermint extract with 500 mL of fresh bedding and each pup was trained over peppermint odor as described previously (McLean et al. 1993). Briefly, odor conditioned pups were stroked with a soft brush vigorously on the rump for 30 sec every other 30 sec for 10 min. The odor only nonlearning control pups were placed on scented bedding for 10 min with no stroking. After the 10 min odor exposure period, pups were returned to the home cage with the dam and other littermates.

Pups were tested by a two-choice odor discrimination test 30 min after the training session in a manner described previously (McLean et al. 1993). Peppermint-scented bedding was prepared as outlined previously and poured into a tray (18 cm × 18 cm); another tray of the same size was filled with 500 mL of fresh bedding. A stainless steel testing box with a 1 cm^2^ wire mesh bottom was placed on the two trays and the trays were positioned 2 cm apart (neutral zone). A fine polypropylene mesh (1 mm^2^ spacing) was placed in the testing box over the wire mesh to allow easy movement of the pups. The testing box and the polypropylene mesh were cleaned with 70% ethanol and allowed to dry prior to use and after testing each pup.

The odor preference test consisted of five 1 min trials. During the testing, the pup was placed in the neutral zone and the timer was started when the pup's head and one paw crossed to either the control or peppermint area. At the end of the minute, the pup was removed from the testing box and given a 1 min rest. The peppermint-bedding tray was closed and the times the pup spent in each area were recorded. On the succeeding trial the pup was placed in the opposite direction (in the neutral zone). The percentage time over peppermint for each pup was calculated by dividing the time spent in the peppermint area over the total activity time (total time spent in both the peppermint and control area). This provided results that normalized litter or individual pup effects so that the level of pup activity did not factor into the calculations. The results of the trials were analyzed using unpaired *t*-tests.

### Decapitation and cryostat sectioning for RNA experiments

Ten minutes after the 10 min test session, pups were decapitated from each litter in the odor/stroke learning group (>50% over peppermint = learning) and the odor nonlearning group (<30% of test time spent over peppermint). The brains were removed from the skull within 2 min, embedded in freezing medium (OCT Tissue-Tek), then flash frozen with 2-methyl-butane slurry for 40 sec and stored at −80°C. Thus, brains from the 30 min testing group were frozen by 52 min posttraining. All instruments for decapitation and the working area were cleaned with RNase Away (Molecular BioProducts), to prevent contamination of the brain tissue by RNases and DNA from the instruments and working area.

Brains were held at −20°C for 24 h prior to cryostat sectioning. The cryostat (Thermo Scientific, Microm HM 550), brushes and chucks used for sectioning were all cleaned with RNase Away; the brains were allowed to acclimatize to the −12°C cryostat temperature for 30 min. Empirical preliminary studies were performed to determine conditions of sacrifice (fixed or unfixed, freezing conditions) and sectioning (temperature, section thickness). Brains were sectioned at 30 µm thickness. The duration of sectioning was limited to 1 h to preserve RNA integrity. The OB sections were put on nuclease- and human nucleic-acid free PEN (polyethylene naphthalate) membrane slides (Leica).

### Quick thionin staining and laser microdissection (LMD)

After cryostat sectioning, the slides were held at 4°C for 10 min before thionin staining. The staining was a quick procedure as suggested by Leica for RNA work with a few modifications. A solution containing 0.5% thionin dissolved in RNase free water was applied and the slides were incubated for 15 sec. Slides were rinsed twice in RNase free water for 15 sec each, once in 70% ethanol for 1 min and once in 90% and 100% ethanol, respectively, for 30 sec each. Slides were then air dried for 10 min at 40°C (on the slide warmer) for 10 min then taken immediately to the LMD microscope (Leica, AS) for cutting of the MCL. All the solutions were prepared with RNase and DNase free water (Life Technologies) and stored in RNase free glass bottles.

The dorsolateral quadrant of the MCL of pups, one from the learning group (odor + stroking) and one from the control group (odor only) from the same litter, were isolated using the LMD microscope (see Fig. 1).

The dorsolateral quadrant was used because this region was shown previously to contain increased pCREB levels when pups were conditioned to peppermint odor on PND6 (McLean et al. 1999). Phosphorylated CREB is critical in odor preference learning (Yuan et al. 2003). The samples were collected by gravity into 0.5 mL VWR microcentrifuge tubes, certified RNase and DNase free. Each of the tubes contained 65 µL of buffer RLT (RNA stabilizing solution and lysis buffer; Qiagen). The total time for LMD was limited to 1 h for each slide. This is because we found longer cutting times resulted in crystallizing of the buffer RLT and RNA of poor quality. Samples were obtained from 20 cryostat sections from both bulbs and included rostral to caudal levels of the bulb, except for the most rostral area, which had poorly defined MCL. The microdissected tissue samples suspended in buffer RLT were stored at −80°C until further isolation of RNA.

### Tissue disruption, homogenization, RNA extraction

The LMD samples in RLT buffer were homogenized by vortexing for 30 sec. The whole OB was homogenized using procedures described in the RNeasy Micro handbook. RNA was then extracted from the microdissected samples using the RNeasy Micro Kit from Qiagen.

### Bioanalyzer analysis

The concentration of RNA extracted from the LMD samples was very low so a bioanalyzer system (Agilent, 2100 Bioanalyzer) was used to evaluate both quantity and quality. The integrity involves the measurement of the ribosomal RNA ratio (the ratio of 28S:18S rRNA) and the relative integrity number (RIN). The RIN has a numeric scale of one to ten with one being RNA of very poor quality and ten being RNA of the highest quality (Agilent, Bioanalyzer 2100 Expert User's Guide, available online). The Agilent RNA 6000 Pico kit was used and the procedures outlined by the manufacturer were followed.

### Microarray and analysis

The microarray analysis of the LMD samples was performed at the microarray facility in the Centre for Applied Genomics, Hospital for Sick Children (Sick Kids) in Toronto, Ontario, Canada. The RNA samples were preamplified with the WT-Ovation One-Direct System (NuGEN Technologies, Inc.) at the microarray facility. Amplification was required because microdissected samples do not have enough RNA for direct microarray. The Rat Gene 1.0 ST Array chip was used to analyze 27,342 genes. 500 picograms (pg) of RNA from each of the samples were used to produce cDNA, which was amplified and 5.5 micrograms (µg) of the cDNA was used for the microarray analysis. A gene differential analysis of the microarray data was first done by the Statistical Analysis Core Facility of the Center for Applied Genomics, Hospital for Sick Children Research Institute, Toronto, but for purposes of the present study, we carried out our own gene differential analysis as described in the Bioinformatics Analysis section below. The following groups were analyzed; the first group (odor + stroking) was treated as the case (experimental) group and the second group (odor only) as the control group.

### Immunohistochemistry experiments

#### Naris occlusion

This study used a within-subject design using naris occlusion. This method involves plugging one naris during training so it will not receive the odor presentation. This will result in no odor reaching the OB ipsilateral to occlusion so odor-related learning would not be expected in that bulb compared to the nonoccluded side. Earlier work demonstrated no difference in lateralized odor preference learning at 6 d as a function of either left or right nares occlusion (Kucharski et al. 1986). For convenience, left nares were used in the IHC experiments. Six pups were used for IHC.

Nose plugs were made out of polyethylene-20 (PE-20) tubing. Silk surgical suture thread (size 5-0) was threaded through the tubing. A piece of human hair was tied around the thread and the thread was knotted around the hair. The knot was pulled into the lumen of the tubing and the ends were trimmed until only about 1 cm of one piece of hair was extended from the tubing. The other end was beveled off for ease of insertion (Cummings et al. 1997).

Prior to training on PND6, each pup was removed the home cage and a small dab of sterile 2% xylocaine gel was placed on its left naris and on the beveled end of the nose plug. The pups were then given one minute of rest before the plugs were inserted gently. The plugs were removed immediately following training. In each litter, one male and one female received training (odor + stroke, O/S)

Ten minutes prior to training, pups were removed from their home cages and placed in a weigh boat for habituation purposes and they received left naris occlusion. The pups were brought to a separate room where they were placed in a small polycarbonate cage containing peppermint-scented bedding (0.3 µL peppermint in 500 ml bedding). The O/S group received stroking with a paintbrush every alternate 30 sec for the 10 min period. During stroking, the pup had to be consistently moving and they were stroked on their back, stomach, and head. The stroking was slightly rough to insure that it was similar to the actions of the dam. After the training period, the nose plugs were removed and the pups were returned to their home cage.

#### Immunohistochemistry

The pups were perfused 50 min following training, in order to duplicate the time period of the microarray analysis. The perfusion procedure was as previously reported by this laboratory (McLean et al. 1999). Brain slices were cut coronally at 30 µm using a cryostat set at −12°C. The brains were transferred to a stage and covered in Tissue Tek. The entire OB was cut from the rostral end to the caudal end, until the cerebral cortex was reached. Following cutting, the slices mounted on microscope slides were left in 4°C for 10 min, followed by room temperature for 10 min. Damage seen in the sections is related to their fragility during the cutting and mounting process and does not reflect premortem injury. The slides were incubated in the primary antibody overnight on a shaker at 4°C. The antibodies used were Dcbld1 (1:1500-1:750, Santa Cruz Biotechnology), Clic2 (1:200, Santa Cruz Biotechnology), Sema4c (1:1000-1:500, Aviva Systems Biology), Magee2 (1:500-1:300, Aviva Systems Biology), Scrg1 (1:500, Aviva Systems Biology), and Sec23b (1:1000, Aviva Systems Biology). The following day the slides were washed using PBS and incubated in a biotinylated secondary antibody to rabbit immunoglobulins (IgG) (Dcbld1, Sema4c, Magee2, Scrg1, and Sec23b) or to goat IgGs (Clic2). The slides were then washed with PBS again and incubated for 1 h in an avidin-biotin-horseradish peroxidase complex (Vectastain ABC kit, Vector Laboratories). In control experiments, antibody was omitted and other steps remained the same. Immunoreactivity was visualized with 0.05% diaminobenzidine in 0.1 M phosphate buffer and 0.01% hydrogen peroxide. Slides were left to dry overnight. The following day they were briefly rehydrated followed by a series of graded alcohols, xylene, and Permount for coverslipping.

Levels of proteins were quantified using relative optical densities (ROD) of region of interest (ROI). The slide images were acquired at 4× objective and even illumination was obtained using Leica camera software. The optical densities (OD) were obtained using Image J software using a 0–255 scale where 0 is black and 255 is white. The ROD of the MCL (and surrounding layers) in the several regions and levels of each bulb were calculated according to the formula ROD = (OD of background − OD of ROI)/OD of background. All sections were analyzed in terms of the glomeruli, EPL, and MCL, except Sec23b which was only analyzed in terms of the MCL, as they were the only cells labeled.

Images were captured using a Leica microscope and Leica digital software and analyzed using ImageJ software. The person analyzing the sections was blind to the treatment side. Every optical density value was calculated by averaging 5 OD values taken from the same area. The background used was usually granule cell or internal plexiform layer containing no visible label. For certain antibodies (cell labeling in the granule cell layer), the olfactory nerve layer or the ependymal zone was used to calculate the OD of the background. The bulb was divided in thirds, providing ∼1 mm rostral, middle, and caudal regions of the OB for analysis. Each section was analyzed separately and as averages (e.g., dorsomedial region average would include rostral, middle, and caudal RODs).

#### Immunohistochemical statistical analysis

All statistical analyses of ROD results were performed by the SPSS. ANOVAs focused on the Hemisphere and Hemisphere × Quadrant conditions that would reflect open and occluded nares differences. When significant ANOVA values were obtained, paired sample *t*-tests were performed on the intra-animal ROD values (occluded versus open naris) for post-hoc analyses.

### Bioinformatics analysis

Expression data from Affymetrix Rat gene 1.0 ST arrays was read into the R environment using the platform design information available in the Bioconductor R package pd.ragene.1.0.st.v1 (version 3.8) and preprocessed using the Robust Multichip Average (RMA) methodology (Irizarry et al. 2003) available in the oligo Bioconductor R package (Carvalho and Irizarry 2010). Probes were mapped to genes using the annotation available from Ensembl BioMart (Kinsella et al. 2011) (release 82) corresponding to the Rat genome assembly 6.0 (GCA_000001895.4). If several probes mapped to a single gene, the expression value for that gene was the average expression of the corresponding probes. In total, for this analysis, expression measurements were calculated for 19,535 Rat genes.

Differentially expressed genes were identified using the local-pooled error (LPE) method (Jain et al. 2003; Park et al. 2007) and *P*-values were corrected for multiple testing using the Benjamini–Hochberg false discovery rate (FDR) method. The LPE method was designed to effectively identify significantly differentially expressed genes with a small number of replicates (Jain et al. 2003). Genes with an absolute log_2_ ratio of at least 1.5 and a FDR-corrected *P*-value of less than 0.05 were deemed to have a statistically significant differentially expression between the two conditions. Using these criteria 24 genes (including protein-coding and nonprotein-coding genes) were found to be differentially expressed. As there has been controversy regarding the fitness of the LPE method being discussed as having reduced power in comparison with other statistical tests of differential expression (Murie et al. 2009), we also carried out the differentially expressed analysis using the Limma method (Smyth 2004) available in the Bioconductor R package Limma. Using Limma t-statistics, no statistically significant differentially expressed gene was found; however, there was a significant Spearman correlation of 0.84 between the fold changes calculated by both approaches.

Gene-set enrichment analysis was performed using the GAGE method (Luo et al. 2009) with the Phenotype, Gene Ontology (Ashburner et al. 2000), and REACTOME (Fabregat et al. 2016) rat gene annotations available in Ensembl Biomart. GAGE was executed with the parameters same.dir set to True and compare set to unpaired. Genes annotated in three GO terms were found to have a concerted expression change in the same direction with a FDR-corrected *P*-value of less than 0.01. These three GO terms were “detection of chemical stimulus involved in sensory perception of smell” (GO:0050911), “olfactory receptor activity” (GO:0004984), and “G-protein coupled receptor activity” (GO:0004930).

## Supplementary Material

Supplemental Material

## References

[LM051177NARC1] Ahmad S, Thulasingam M, Palombo I, Daley DO, Johnson KA, Morgenstern R, Haeggström JZ, Rinaldo-Matthis A. 2015 Trimeric microsomal glutathione transferase 2 displays one third of the sites reactivity. Biochim Biophys Acta 1854: 1365–1371. 10.1016/j.bbapap.2015.06.003 26066610

[LM051177NARC2] Aomatsu E, Takahashi N, Sawada S, Okubo N, Hasegawa T, Taira M, Miura H, Ishisaki A, Chosa N. 2014 Novel SCRG1/BST1 axis regulates self-renewal, migration, and osteogenic differentiation potential in mesenchymal stem cells. Sci Rep 4: 3652 10.1038/srep03652 24413464 PMC3888969

[LM051177NARC3] Ashburner M, Ball CA, Blake JA, Botstein D, Butler H, Cherry JM, Davis AP, Dolinski K, Dwight SS, Eppig JT, 2000 Gene ontology: tool for the unification of biology. The Gene Ontology Consortium. Nat Genet 25: 25–29. 10.1038/75556 10802651 PMC3037419

[LM051177NARC4] Augustin H, McGourty K, Steinert JR, Cocheme HM, Adcott J, Cabecinha M, Vincent A, Halff EF, Kittler JT, Boucrot E, 2017 Myostatin-like proteins regulate synaptic function and neuronal morphology. Development 144: 2445–2455. 10.1242/dev.152975 28533206 PMC5536874

[LM051177NARC5] Autio KJ, Kastaniotis AJ, Pospiech H, Miinalainen IJ, Schonauer MS, Dieckmann CL, Hiltunen JK. 2008 An ancient genetic link between vertebrate mitochondrial fatty acid synthesis and RNA processing. FASEB J 22: 569–578. 10.1096/fj.07-8986 17898086

[LM051177NARC6] Bhattacharya S, Mukherjee B, Doré JJE, Yuan Q, Harley CW, McLean JH. 2017 Histone deacetylase inhibition induces odor preference memory extension and maintains enhanced AMPA receptor expression in the rat pup model. Learn Mem 24: 543–551. 10.1101/lm.045799.117 28916629 PMC5602343

[LM051177NARC7] Brillantes AB, Ondrias K, Scott A, Kobrinsky E, Ondriašová E, Moschella MC, Jayaraman T, Landers M, Ehrlich BE, Marks AR. 1994 Stabilization of calcium release channel (ryanodine receptor) function by FK506-binding protein. Cell 77: 513–523. 10.1016/0092-8674(94)90214-3 7514503

[LM051177NARC8] Brunelli M, Castellucci V, Kandel ER. 1976 Synaptic facilitation and behavioral sensitization in *Aplysia*: possible role of serotonin and cyclic AMP. Science 194: 1178–1181. 10.1126/science.186870 186870

[LM051177NARC9] Carvalho BS, Irizarry RA. 2010 A framework for oligonucleotide microarray preprocessing. Bioinformatics 26: 2363–2367. 10.1093/bioinformatics/btq431 20688976 PMC2944196

[LM051177NARC10] Christie-Fougere MM, Darby-King A, Harley CW, McLean JH. 2009 Calcineurin inhibition eliminates the normal inverted U curve, enhances acquisition and prolongs memory in a mammalian 3′–5′-cyclic AMP-dependent learning paradigm. Neuroscience 158: 1277–1283. 10.1016/j.neuroscience.2008.11.004 19041926

[LM051177NARC11] Cui W, Smith A, Darby-King A, Harley CW, McLean JH. 2007 A temporal-specific and transient cAMP increase characterizes odorant classical conditioning. Learn Mem 14: 126–133. 10.1101/lm.496007 17337703 PMC1838553

[LM051177NARC12] Cui W, Darby-King A, Grimes MT, Howland JG, Wang YT, McLean JH, Harley CW. 2011 Odor preference learning and memory modify GluA1 phosphorylation and GluA1 distribution in the neonate rat olfactory bulb: testing the AMPA receptor hypothesis in an appetitive learning model. Learn Mem 18: 283–291. 10.1101/lm.1987711 21498562

[LM051177NARC13] Cui-Wang T, Hanus C, Cui T, Helton T, Bourne J, Watson D, Harris KM, Ehlers MD. 2012 Local zones of endoplasmic reticulum complexity confine cargo in neuronal dendrites. Cell 148: 309–321. 10.1016/j.cell.2011.11.056 22265418 PMC3266556

[LM051177NARC14] Cummings DM, Henning HE, Brunjes PC. 1997 Olfactory bulb recovery after early sensory deprivation. J Neurosci 17: 7433–7440. 10.1523/JNEUROSCI.17-19-07433.1997 9295389 PMC6573448

[LM051177NARC15] Dandoy-Dron F, Griffond B, Mishal Z, Tovey MG, Dron M. 2003 Scrg1, a novel protein of the CNS is targeted to the large dense-core vesicles in neuronal cells. Eur J Neurosci 18: 2449–2459. 10.1046/j.1460-9568.2003.03009.x 14622145

[LM051177NARC16] De Donato M, Peters SO, Hussain T, Rodulfo H, Thomas BN, Babar ME, Imumorin IG. 2017 Molecular evolution of type II MAGE genes from ancestral MAGED2 gene and their phylogenetic resolution of basal mammalian clades. Mamm Genome 28: 443–454. 10.1007/s00335-017-9695-6 28516231

[LM051177NARC17] Doyle JM, Gao J, Wang J, Yang M, Potts PR. 2010 MAGE-RING protein complexes comprise a family of E3 ubiquitin ligases. Mol Cell 39: 963–974. 10.1016/j.molcel.2010.08.029 20864041 PMC4509788

[LM051177NARC18] Dron M, Dandoy-Dron F, Guillo F, Benboudjema L, Hauw JJ, Lebon P, Dormont D, Tovey MG. 1998 Characterization of the human analogue of a Scrapie-responsive gene. J Biol Chem 273: 18015–18018. 10.1074/jbc.273.29.18015 9660755

[LM051177NARC19] Dron M, Tartare X, Guillo F, Haik S, Barbin G, Maury C, Tovey M, Dandoy-Dron F. 2000 Mouse scrapie responsive gene 1 (Scrg1): genomic organization, physical linkage to sap30, genetic mapping on chromosome 8, and expression in neuronal primary cell cultures. Genomics 70: 140–149. 10.1006/geno.2000.6358 11087671

[LM051177NARC20] Dron M, Bailly Y, Beringue V, Haeberlé AM, Griffond B, Risold PY, Tovey MG, Laude H, Dandoy-Dron F. 2005 Scrg1 is induced in TSE and brain injuries, and associated with autophagy. Eur J Neurosci 22: 133–146. 10.1111/j.1460-9568.2005.04172.x 16029203

[LM051177NARC21] Dulhunty AF, Pouliquin P, Coggan M, Gage PW, Board PG. 2005 A recently identified member of the glutathione transferase structural family modifies cardiac RyR2 substate activity, coupled gating and activation by Ca^2+^ and ATP. Biochem J 390: 333–343. 10.1042/BJ20042113 15916532 PMC1184587

[LM051177NARC22] Evans AJ, Gurung S, Wilkinson KA, Stephens DJ, Henley JM. 2017 Assembly, secretory pathway trafficking, and surface delivery of kainate receptors is regulated by neuronal activity. Cell Rep 19: 2613–2626. 10.1016/j.celrep.2017.06.001 28636947 PMC5489663

[LM051177NARC23] Fabregat A, Sidiropoulos K, Garapati P, Gillespie M, Hausmann K, Haw R, Jassal B, Jupe S, Korninger F, McKay S, 2016 The Reactome pathway knowledgebase. Nucleic Acids Res 44: D481–D487. 10.1093/nar/gkv1351 26656494 PMC4702931

[LM051177NARC24] Fang X, Robinson J, Wang-Hu J, Jiang L, Freeman DA, Rivkees SA, Wendler CC. 2015 cAMP induces hypertrophy and alters DNA methylation in HL-1 cardiomyocytes. Am J Physiol Cell Physiol 309: C425–C436. 10.1152/ajpcell.00058.2015 26224577 PMC4572371

[LM051177NARC25] Fillion TJ, Blass EM. 1986 Infantile experience with suckling odors determines adult sexual behavior in male rats. Science 231: 729–731. 10.1126/science.3945807 3945807

[LM051177NARC26] Fontaine CJ, Harley CW, Yuan Q. 2013 Lateralized odor preference training in rat pups reveals an enhanced network response in anterior piriform cortex to olfactory input that parallels extended memory. J Neurosci 33: 15126–15131. 10.1523/JNEUROSCI.2503-13.2013 24048843 PMC6618408

[LM051177NARC27] Gavazzi I. 2001 Semaphorin-neuropilin-1 interactions in plasticity and regeneration of adult neurons. Cell Tissue Res 305: 275–284. 11545265 10.1007/s004410100365

[LM051177NARC28] Grimes MT, Smith M, Li X, Darby-King A, Harley CW, McLean JH. 2011 Mammalian intermediate-term memory: new findings in neonate rat. Neurobiol Learn Mem 95: 385–391. 10.1016/j.nlm.2011.01.012 21296674

[LM051177NARC29] Grimes MT, Harley CW, Darby-King A, McLean JH. 2012 PKA increases in the olfactory bulb act as unconditioned stimuli and provide evidence for parallel memory systems: pairing odor with increased PKA creates intermediate- and long-term, but not short-term, memories. Learn Mem 19: 107–115. 10.1101/lm.024489.111 22354948

[LM051177NARC30] Hasegawa K, Yasuda T, Shiraishi C, Fujiwara K, Przedborski S, Mochizuki H, Yoshikawa K. 2016 Promotion of mitochondrial biogenesis by necdin protects neurons against mitochondrial insults. Nat Commun 7: 10943 10.1038/ncomms10943 26971449 PMC4793078

[LM051177NARC31] Irizarry RA, Bolstad BM, Collin F, Cope LM, Hobbs B, Speed TP. 2003 Summaries of Affymetrix GeneChip probe level data. Nucleic Acids Res 31: e15 10.1093/nar/gng015 12582260 PMC150247

[LM051177NARC32] Iwasaki S, Miyake M, Watanabe H, Kitagawa E, Watanabe K, Ohwada S, Kitazawa H, Rose MT, Aso H. 2013 Expression of myostatin in neural cells of the olfactory system. Mol Neurobiol 47: 1–8. 10.1007/s12035-012-8342-1 22941030

[LM051177NARC33] Iyer SC, Ramachandran Iyer EP, Meduri R, Rubaharan M, Kuntimaddi A, Karamsetty M, Cox DN. 2013 Cut, via CrebA, transcriptionally regulates the COPII secretory pathway to direct dendrite development in Drosophila. J Cell Sci 126: 4732–4745. 10.1242/jcs.131144 23902691 PMC3795340

[LM051177NARC34] Jain N, Thatte J, Braciale T, Ley K, O'Connell M, Lee JK. 2003 Local-pooled-error test for identifying differentially expressed genes with a small number of replicated microarrays. Bioinformatics 19: 1945–1951. 10.1093/bioinformatics/btg264 14555628

[LM051177NARC35] Jerome D, Hou Q, Yuan Q. 2012 Interaction of NMDA receptors and L-type calcium channels during early odor preference learning in rats. Eur J Neurosci 36: 3134–3141. 10.1111/j.1460-9568.2012.08210.x 22762736

[LM051177NARC36] Jiang T, Altman S. 2002 A protein subunit of human RNase P, Rpp14, and its interacting partner, OIP2, have 3′→5′ exoribonuclease activity. Proc Natl Acad Sci 99: 5295–5300. 11929972 10.1073/pnas.072083699 PMC122763

[LM051177NARC37] Kandel ER. 2012 The molecular biology of memory: cAMP, PKA, CRE, CREB-1, CREB-2, and CPEB. Mol Brain 5: 14 10.1186/1756-6606-5-14 22583753 PMC3514210

[LM051177NARC38] Khoriaty R, Hesketh GG, Bernard A, Weyand AC, Mellacheruvu D, Zhu G, Hoenerhoff MJ, McGee B, Everett L, Adams EJ, 2018 Functions of the COPII gene paralogs SEC23A and SEC23B are interchangeable in vivo. Proc Natl Acad Sci 115: E7748–E7757. 10.1073/pnas.1805784115 30065114 PMC6099849

[LM051177NARC39] Kiltschewskij D, Cairns MJ. 2019 Temporospatial guidance of activity-dependent gene expression by microRNA: mechanisms and functional implications for neural plasticity. Nucleic Acids Res 47: 533–545. 10.1093/nar/gky1235 30535081 PMC6344879

[LM051177NARC40] Kim S, Chiba A. 2004 Dendritic guidance. Trends Neurosci 27: 194–202. 10.1016/j.tins.2004.02.011 15046878

[LM051177NARC41] Kimura F, Nakamura S. 1985 Locus coeruleus neurons in the neonatal rat: electrical activity and responses to sensory stimulation. Brain Res 355: 301–305. 10.1016/0165-3806(85)90055-0 4084787

[LM051177NARC42] Kinsella RJ, Kahari A, Haider S, Zamora J, Proctor G, Spudich G, Almeida-King J, Staines D, Derwent P, Kerhornou A, 2011 Ensembl BioMarts: a hub for data retrieval across taxonomic space. Database (Oxford) 2011: bar030 10.1093/database/bar030 21785142 PMC3170168

[LM051177NARC43] Korneev SA, Vavoulis DV, Naskar S, Dyakonova VE, Kemenes I, Kemenes G. 2018 A CREB2-targeting microRNA is required for long-term memory after single-trial learning. Sci Rep 8: 3950 10.1038/s41598-018-22278-w 29500383 PMC5834643

[LM051177NARC44] Kou CH, Zhou T, Han XL, Zhuang HJ, Qian HX. 2016 Downregulation of mir-23b in plasma is associated with poor prognosis in patients with colorectal cancer. Oncol Lett 12: 4838–4844. 10.3892/ol.2016.5265 28101227 PMC5228308

[LM051177NARC45] Kucharski D, Johanson IB, Hall WG. 1986 Unilateral olfactory conditioning in 6-day-old rat pups. Behav Neural Biol 46: 472–490. 10.1016/S0163-1047(86)90506-6 3814049

[LM051177NARC46] Kukushkin NV, Carew TJ. 2017 Memory takes time. Neuron 95: 259–279. 10.1016/j.neuron.2017.05.029 28728021 PMC6053684

[LM051177NARC47] Lee KF, Soares C, Thivierge JP, Béïque JC. 2016 Correlated synaptic inputs drive dendritic calcium amplification and cooperative plasticity during clustered synapse development. Neuron 89: 784–799. 10.1016/j.neuron.2016.01.012 26853305

[LM051177NARC48] Lein ES, Hawrylycz MJ, Ao N, Ayres M, Bensinger A, Bernard A, Boe AF, Boguski MS, Brockway KS, Byrnes EJ, 2007 Genome-wide atlas of gene expression in the adult mouse brain. Nature 445: 168–176. 10.1038/nature05453 17151600

[LM051177NARC49] Lethbridge R, Hou Q, Harley CW, Yuan Q. 2012 Olfactory bulb glomerular NMDA receptors mediate olfactory nerve potentiation and odor preference learning in the neonate rat. PloS One 7: e35024 10.1371/journal.pone.0035024 22496886 PMC3319620

[LM051177NARC50] Li Y, Li Z, Zhou S, Wen J, Geng B, Yang J, Cui Q. 2013 Genome-wide analysis of human microRNA stability. Biomed Res Int 2013: 368975 10.1155/2013/368975 24187663 PMC3804285

[LM051177NARC51] Li H, Wu C, Aramayo R, Sachs MS, Harlow ML. 2015 Synaptic vesicles contain small ribonucleic acids (sRNAs) including transfer RNA fragments (trfRNA) and microRNAs (miRNA). Sci Rep 5: 14918 10.1038/srep14918 26446566 PMC4597359

[LM051177NARC52] Luo W, Friedman MS, Shedden K, Hankenson KD, Woolf PJ. 2009 GAGE: generally applicable gene set enrichment for pathway analysis. BMC Bioinformatics 10: 161 10.1186/1471-2105-10-161 19473525 PMC2696452

[LM051177NARC53] Majid S, Dar AA, Saini S, Arora S, Shahryari V, Zaman MS, Chang I, Yamamura S, Tanaka Y, Deng G, 2012 miR-23b represses proto-oncogene Src kinase and functions as methylation-silenced tumor suppressor with diagnostic and prognostic significance in prostate cancer. Cancer Res 72: 6435–6446. 10.1158/0008-5472.CAN-12-2181 23074286 PMC3940348

[LM051177NARC54] McCaughey J, Stephens DJ. 2018 COPII-dependent ER export in animal cells: adaptation and control for diverse cargo. Histochem Cell Biol 150: 119–131. 10.1007/s00418-018-1689-2 29916038 PMC6096569

[LM051177NARC55] McLean JH, Darby-King A, Sullivan RM, King SR. 1993 Serotonergic influence on olfactory learning in the neonate rat. Behav Neural Biol 60: 152–162. 10.1016/0163-1047(93)90257-I 7906939

[LM051177NARC56] McLean JH, Harley CW, Darby-King A, Yuan Q. 1999 pCREB in the neonate rat olfactory bulb is selectively and transiently increased by odor preference-conditioned training. Learn Mem 6: 608–618. 10.1101/lm.6.6.608 10641765 PMC311313

[LM051177NARC57] McLean JH, Darby-King A, Harley CW. 2005 Potentiation and prolongation of long-term odor memory in neonate rats using a phosphodiesterase inhibitor. Neuroscience 135: 329–334. 10.1016/j.neuroscience.2005.06.029 16111826

[LM051177NARC58] Meng X, Wang G, Viero C, Wang Q, Mi W, Su XD, Wagenknecht T, Williams AJ, Liu Z, Yin CC. 2009 CLIC2-RyR1 interaction and structural characterization by cryo-electron microscopy. J Mol Biol 387: 320–334. 10.1016/j.jmb.2009.01.059 19356589 PMC2667806

[LM051177NARC59] Modarresi S, Mukherjee B, McLean JH, Harley CW, Yuan Q. 2016 CaMKII mediates stimulus specificity in early odor preference learning in rats. J Neurophysiol 116: 404–410. 10.1152/jn.00176.2016 27121578 PMC4969389

[LM051177NARC60] Murie C, Woody O, Lee AY, Nadon R. 2009 Comparison of small n statistical tests of differential expression applied to microarrays. BMC Bioinformatics 10: 45 10.1186/1471-2105-10-45 19192265 PMC2674054

[LM051177NARC61] Nakamura S, Kimura F, Sakaguchi T. 1987 Postnatal development of electrical activity in the locus ceruleus. J Neurophysiol 58: 510–524. 10.1152/jn.1987.58.3.510 3655880

[LM051177NARC62] Ng D, Pitcher GM, Szilard RK, Sertié A, Kanisek M, Clapcote SJ, Lipina T, Kalia LV, Joo D, McKerlie C, 2009 Neto1 is a novel CUB-domain NMDA receptor-interacting protein required for synaptic plasticity and learning. PLoS Biol 7: e41 10.1371/journal.pbio.1000041 19243221 PMC2652390

[LM051177NARC63] Padua RA, Yamamoto T, Fyda D, Sawchuk MA, Geiger JD, Nagy JI. 1992 Autoradiographic analysis of [3H]Ryanodine binding sites in rat brain: regional distribution and the effects of lesions on sites in the hippocampus. J Chem Neuroanat 5: 63–73. 10.1016/0891-0618(92)90034-N 1605914

[LM051177NARC64] Pandipati S, Schoppa NE. 2012 Age-dependent adrenergic actions in the main olfactory bulb that could underlie an olfactory-sensitive period. J Neurophysiol 108: 1999–2007. 10.1152/jn.00322.2012 22815401 PMC3545001

[LM051177NARC65] Park T, Kim Y, Bekiranov S, Lee JK. 2007 Error-pooling-based statistical methods for identifying novel temporal replication profiles of human chromosomes observed by DNA tiling arrays. Nucleic Acids Res 35: e69 10.1093/nar/gkm130 17430969 PMC1888820

[LM051177NARC66] Pellegrino L, Krell J, Roca-Alonso L, Stebbing J, Castellano L. 2013 MicroRNA-23b regulates cellular architecture and impairs motogenic and invasive phenotypes during cancer progression. Bioarchitecture 3: 119–124. 10.4161/bioa.26134 24002530 PMC4201606

[LM051177NARC67] Pennypacker KR, Hudson PM, Hong JS, McMillian MK. 1995 DNA binding activity of CREB transcription factors during ontogeny of the central nervous system. Brain Res Dev Brain Res 86: 242–249. 10.1016/0165-3806(95)00033-A 7656416

[LM051177NARC68] Pick JE, Khatri L, Sathler MF, Ziff EB. 2017 mGluR long-term depression regulates GluA2 association with COPII vesicles and exit from the endoplasmic reticulum. EMBO J 36: 232–244. 10.15252/embj.201694526 27856517 PMC5239995

[LM051177NARC69] Rangaraju V, Tom Dieck S, Schuman EM. 2017 Local translation in neuronal compartments: how local is local? EMBO Rep 18: 693–711. 10.15252/embr.201744045 28404606 PMC5412868

[LM051177NARC70] Sanhueza M, Lisman J. 2013 The CaMKII/NMDAR complex as a molecular memory. Mol Brain 6: 10 10.1186/1756-6606-6-10 23410178 PMC3582596

[LM051177NARC71] Schmoker AM, Ebert AM, Ballif BA. 2019 The DCBLD receptor family: emerging signaling roles in development, homeostasis and disease. Biochem J 476: 931–950. 10.1042/BCJ20190022 30902898

[LM051177NARC72] Shah A, Oxley G, Lovic V, Fleming AS. 2002 Effects of preweaning exposure to novel maternal odors on maternal responsiveness and selectivity in adulthood. Dev Psychobiol 41: 187–196. 10.1002/dev.10064 12325133

[LM051177NARC73] Shakhawat AM, Gheidi A, Hou Q, Dhillon SK, Marrone DF, Harley CW, Yuan Q. 2014 Visualizing the engram: learning stabilizes odor representations in the olfactory network. J Neurosci 34: 15394–15401. 10.1523/JNEUROSCI.3396-14.2014 25392506 PMC6608445

[LM051177NARC74] Smalheiser NR. 2014 The RNA-centred view of the synapse: non-coding RNAs and synaptic plasticity. Philos Trans R Soc Lond B Biol Sci 369: 20130504 10.1098/rstb.2013.0504 25135965 PMC4142025

[LM051177NARC75] Smyth GK. 2004 Linear models and empirical bayes methods for assessing differential expression in microarray experiments. Stat Appl Genet Mol Biol 3: Article3 10.2202/1544-6115.1027 16646809

[LM051177NARC76] Sullivan RM, Leon M. 1986 Early olfactory learning induces an enhanced olfactory bulb response in young rats. Brain Res 392: 278–282. 10.1016/0165-3806(86)90256-7 3708381

[LM051177NARC77] Sullivan RM, Leon M. 1987 One-trial olfactory learning enhances olfactory bulb responses to an appetitive conditioned odor in 7-day-old rats. Brain Res 432: 307–311. 10.1016/0165-3806(87)90056-3 3676845

[LM051177NARC78] Sullivan RM, Wilson DA, Leon M. 1989 Norepinephrine and learning-induced plasticity in infant rat olfactory system. J Neurosci 9: 3998–4006. 10.1523/JNEUROSCI.09-11-03998.1989 2585063 PMC1885972

[LM051177NARC79] Widagdo J, Guntupalli S, Jang SE, Anggono V. 2017 Regulation of AMPA receptor trafficking by protein ubiquitination. Front Mol Neurosci 10: 347 10.3389/fnmol.2017.00347 29123470 PMC5662755

[LM051177NARC80] Wiegert JS, Pulin M, Gee CE, Oertner TG. 2018 The fate of hippocampal synapses depends on the sequence of plasticity-inducing events. Elife 7: e39151 10.7554/eLife.39151 30311904 PMC6205809

[LM051177NARC81] Ye B, Zhang Y, Song W, Younger SH, Jan LY, Jan YN. 2007 Growing dendrites and axons differ in their reliance on the secretory pathway. Cell 130: 717–729. 10.1016/j.cell.2007.06.032 17719548 PMC2020851

[LM051177NARC82] Yuan Q, Harley CW, McLean JH, Knöpfel T. 2002 Optical imaging of odor preference memory in the rat olfactory bulb. J Neurophysiol 87: 3156–3159. 10.1152/jn.00917.2001 12037216

[LM051177NARC83] Yuan Q, Harley CW, Darby-King A, Neve RL, McLean JH. 2003 Early odor preference learning in the rat: bidirectional effects of cAMP response element-binding protein (CREB) and mutant CREB support a causal role for phosphorylated CREB. J Neurosci 23: 4760–4765. 10.1523/JNEUROSCI.23-11-04760.2003 12805315 PMC6740781

[LM051177NARC84] Yuan Q, Shakhawat AM, Harley CW. 2014 Mechanisms underlying early odor preference learning in rats. Prog Brain Res 208: 115–156. 10.1016/B978-0-444-63350-7.00005-X 24767481

[LM051177NARC85] Zhou P, Zhao YT, Guo YB, Xu SM, Bai SH, Lakatta EG, Cheng H, Hao XM, Wang SQ. 2009 β-adrenergic signaling accelerates and synchronizes cardiac ryanodine receptor response to a single L-type Ca^2+^ channel. Proc Natl Acad Sci 106: 18028–18033. 10.1073/pnas.0906560106 19815510 PMC2758811

[LM051177NARC86] Zhu M, Tao J, Vasievich MP, Wei W, Zhu G, Khoriaty RN, Zhang B. 2015 Neural tube opening and abnormal extraembryonic membrane development in SEC23A deficient mice. Sci Rep 5: 15471 10.1038/srep15471 26494538 PMC4616029

[LM051177NARC87] Zuloaga R, Fuentes EN, Molina A, Valdés JA. 2013 The cAMP response element binding protein (CREB) is activated by insulin-like growth factor-1 (IGF-1) and regulates myostatin gene expression in skeletal myoblast. Biochem Biophys Res Commun 440: 258–264. 10.1016/j.bbrc.2013.09.067 24064350

