# Supplementary material for: Learning-induced mRNA alterations in olfactory bulb mitral cells in neonatal rats
Source: Learn Mem. 2020 May;27(5):209–21. doi: 10.1101/lm.051177.119 (PMC7164515; doi:10.1101/lm.051177.119)
Supplement: Supplemental Material [file supp_27_5_209__index.html]

Supplemental Material 

# Learning-induced mRNA alterations in olfactory bulb mitral cells in neonatal rats

## Supplemental Material

- Supplementary\_Figure1.png
- Supplemental\_Spreadsheet\_1.xls
- Supplemental\_Spreadsheet\_2.xls
