# Supplementary figures and images for: Learning-induced mRNA alterations in olfactory bulb mitral cells in neonatal rats
Source: Learn Mem. 2020 May;27(5):209–21. doi: 10.1101/lm.051177.119 (PMC7164515; doi:10.1101/lm.051177.119)

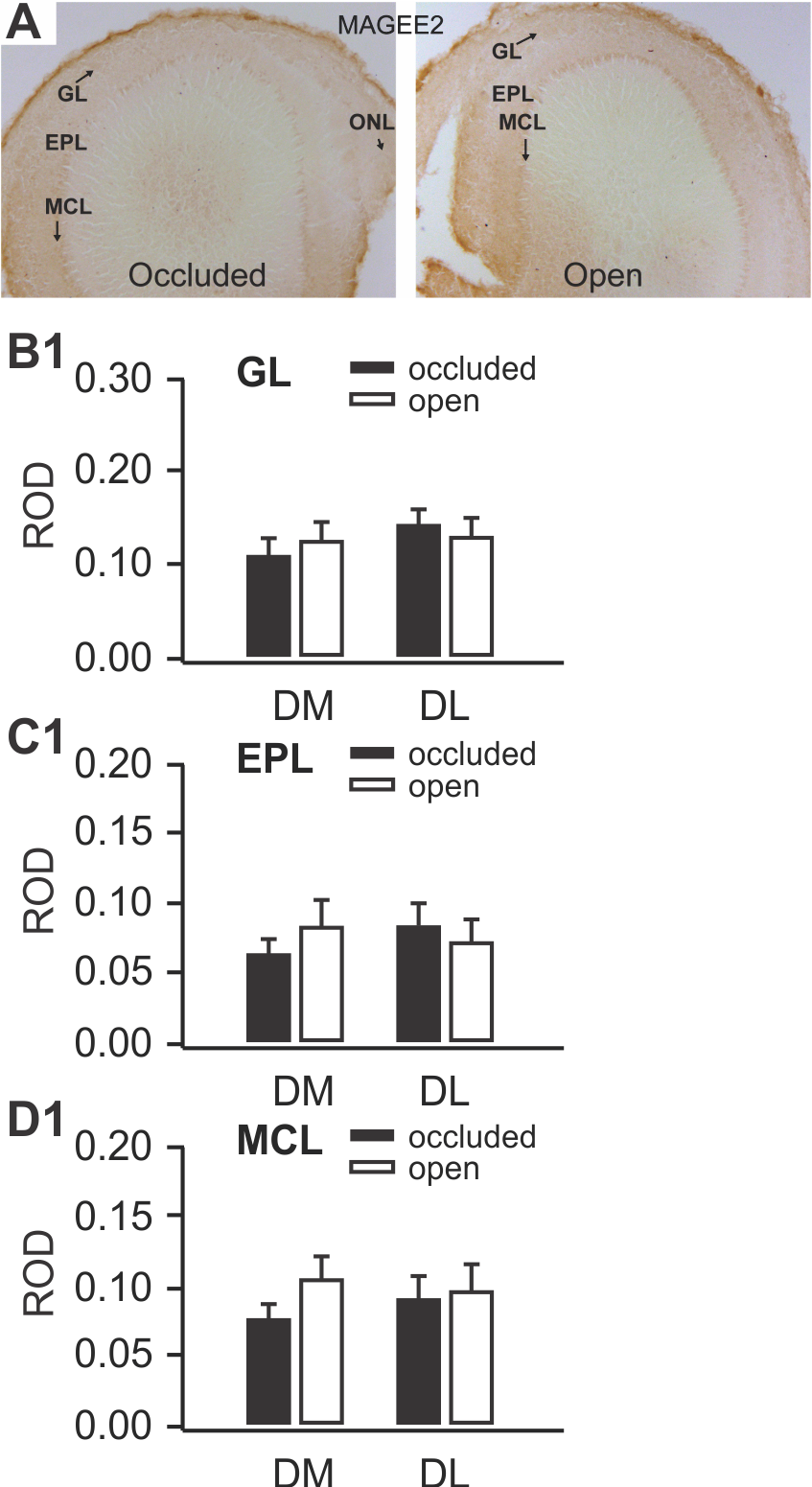

Supplement: Supplemental Material [file supp_27.5.209_Supplementary_Figure1.png]
